# Unmasking Risk in Mitral Regurgitation: Prognostic Value of Exercise Stress Echocardiography—A Systematic Review

**DOI:** 10.3390/jcm15093253

**Published:** 2026-04-24

**Authors:** Andrea Sonaglioni, Massimo Baravelli, Giulio Francesco Gramaglia, Gian Luigi Nicolosi, Michele Lombardo

**Affiliations:** 1 Division of Cardiology, IRCCS MultiMedica, 20123 Milan, Italy; massimo.baravelli@multimedica.it (M.B.); michele.lombardo@multimedica.it (M.L.); 2 Department of Emergency, Fondazione IRCSS Ca’ Granda, Ospedale Maggiore Policlinico, 20122 Milan, Italy; giulio.gramaglia@unimi.it; 3 Division of Cardiology, Policlinico San Giorgio, 33170 Pordenone, Italy; gianluigi.nicolosi@gmail.com

**Keywords:** mitral regurgitation, exercise stress echocardiography, risk stratification, asymptomatic patients, prognosis

## Abstract

**Background:** Risk stratification of patients with mitral regurgitation (MR), including both primary (degenerative) and secondary (functional) forms, remains challenging, particularly in asymptomatic or minimally symptomatic stages, as clinical assessment and resting echocardiography may underestimate disease severity and functional impairment. Exercise stress echocardiography (ESE) enables dynamic evaluation of regurgitation severity, ventricular performance, and cardiopulmonary response, potentially improving prognostic assessment. **Methods:** A systematic review was conducted according to PRISMA guidelines. PubMed, Scopus, and EMBASE were searched from inception to March 2026. Studies including adult patients with primary or secondary MR undergoing exercise-based stress echocardiography and reporting clinical outcomes were selected. Studies using exclusively pharmacological stress were excluded. Data were qualitatively synthesized, and continuous variables were summarized as weighted medians and interquartile ranges. In addition, emerging and non-conventional prognostic markers, including anatomical indices such as the modified Haller index (MHI), were explored to provide a more comprehensive risk stratification framework. **Results:** Nineteen studies were included, encompassing a heterogeneous population in terms of MR etiology, severity, and clinical presentation. During follow-up, a substantial proportion of patients experienced adverse events, including heart failure, mitral valve intervention, or death. Exercise-derived parameters consistently showed strong prognostic value. In particular, exercise-induced worsening of MR severity (increase in effective regurgitant orifice area and regurgitant volume), absence of contractile reserve, elevated filling pressures (E/e’), and exercise-induced pulmonary hypertension were associated with worse outcomes. Reduced functional capacity and impaired right ventricular–pulmonary arterial coupling provided additional prognostic information. Emerging markers, including chest wall configuration assessed by MHI, appeared to further refine risk stratification in selected patient subsets. In contrast, resting parameters were less consistently predictive. **Conclusions:** ESE provides incremental prognostic information in patients with MR by identifying dynamic abnormalities not evident at rest. Its integration into clinical evaluation, together with novel anatomical and functional markers, may improve risk stratification and support earlier identification of high-risk patients who could benefit from timely intervention. Further studies are needed to standardize methodologies and define clinically relevant thresholds.

## 1. Introduction

Mitral regurgitation (MR) is one of the most prevalent valvular heart diseases worldwide and represents a major contributor to cardiovascular morbidity and mortality [1]. Its clinical spectrum is highly heterogeneous, encompassing both primary (degenerative) and secondary (functional) forms, with different underlying mechanisms but often overlapping clinical trajectories [2]. Despite advances in imaging and therapeutic strategies, the optimal timing of intervention—particularly in patients with asymptomatic or minimally symptomatic MR—remains a critical and unresolved clinical issue [3].

The natural history of MR is characterized by a prolonged compensated phase, during which patients may remain clinically stable despite progressive structural and functional alterations [4]. However, this apparent stability can be misleading. Subclinical left ventricular (LV) dysfunction, progressive volume overload, and increasing pulmonary pressures may develop before overt symptoms become evident [5]. As a result, reliance on symptom status alone may delay referral for intervention, potentially exposing patients to irreversible myocardial damage and worse postoperative outcomes [6].

In this context, there is a growing need for objective tools capable of detecting early functional impairment and refining risk stratification beyond conventional resting echocardiographic parameters [7,8]. While resting measures such as left ventricular ejection fraction (LVEF), effective regurgitant orifice area (EROA), and regurgitant volume are central to disease grading, they provide only a static snapshot of a dynamic condition [9]. Importantly, MR severity and its hemodynamic consequences are highly load-dependent and may change significantly under physiological stress [10].

Exercise stress echocardiography (ESE) offers a unique opportunity to evaluate MR within a dynamic framework. By integrating hemodynamic, functional, and structural information during exercise, ESE allows real-time assessment of regurgitation severity, ventricular performance, and cardiopulmonary interaction [11]. In particular, stress-induced changes in EROA and regurgitant volume, elevation of LV filling pressures (e.g., E/e’), and increases in pulmonary artery pressure provide insight into the functional reserve of the cardiovascular system [12]. Additionally, indices of myocardial deformation such as global longitudinal strain (GLS) [13], as well as markers of right ventricular (RV)–pulmonary arterial (PA) coupling [e.g., tricuspid annular plane systolic excursion/systolic pulmonary artery pressure (TAPSE/sPAP) ratio] [14], further contribute to a comprehensive evaluation of disease impact.

Over the past two decades, a substantial number of studies have explored the prognostic implications of ESE in MR across different clinical settings, including asymptomatic degenerative MR and functional MR associated with LV dysfunction [15]. These investigations have identified a wide array of potential predictors, ranging from exercise-induced worsening of regurgitation to impaired contractile reserve and abnormal hemodynamic responses. Notably, dynamic parameters such as exercise EROA, increase in regurgitant volume, elevated E/e’, and exercise pulmonary hypertension have consistently emerged as markers associated with adverse clinical outcomes [16].

At the same time, clinical and functional variables—including age, atrial fibrillation, reduced exercise capacity, and biomarkers such as natriuretic peptides—have been shown to provide complementary prognostic information [17,18]. This highlights the multifactorial nature of risk in MR and the importance of integrating imaging findings with clinical context. However, the heterogeneity in study design, patient populations, stress protocols, and endpoint definitions has limited the generalizability of individual findings and hindered their translation into routine clinical practice. Moreover, the populations evaluated in previous studies are diverse, encompassing patients with preserved or reduced LVEF, primary and secondary MR, and varying degrees of symptom burden. This heterogeneity, while reflecting real-world clinical practice, further underscores the need for a structured synthesis of the available evidence.

Given these considerations, a comprehensive and systematic evaluation of the prognostic role of ESE in MR is warranted. The present systematic review aims to provide an integrated overview of the available literature, focusing on exercise-derived echocardiographic and hemodynamic parameters associated with clinical outcomes. In addition, this work seeks to characterize the clinical profile of the studied populations and to describe the physiological response to exercise in MR, in order to better define the role of ESE in contemporary risk stratification and clinical decision-making.

## 2. Materials and Methods

This systematic review was performed in line with the Preferred Reporting Items for Systematic Reviews and Meta-Analyses (PRISMA) guidelines [19] (Supplementary Materials File S1). The protocol was prospectively registered in April 2026 on the International Platform of Registered Systematic Review and Meta-analysis Protocols (INPLASY) [1,2,3,4,5,6,7,8,9,10,11,12,13,14,15,16,17,18] (registration number: INPLASY202640023; registration date: 7 April 2026; Supplementary Materials File S2). The study design, inclusion criteria, and analytical approach were established in advance and applied consistently throughout all stages of the review.

### 2.1. Search Strategy

A comprehensive literature search was independently performed by two investigators to identify studies evaluating the prognostic role of ESE in patients with MR. Studies enrolling patients with both primary (degenerative) and secondary (functional) MR were considered eligible, in order to reflect the broad clinical spectrum of the disease. Electronic databases, including PubMed, Scopus, and EMBASE, were systematically searched from database inception to March 2026.

The search strategy combined controlled vocabulary and free-text terms related to MR and stress echocardiography, including “mitral regurgitation”, “exercise stress echocardiography”, “stress echo”, “exercise testing”, “hemodynamics”, “prognosis”, “outcome”, and “risk stratification”. Search results were screened without restriction to MR etiology. No restrictions were applied regarding language, publication date, or geographic setting.

In addition to the electronic search, the reference lists of all included studies and relevant review articles were manually screened to identify further eligible publications. Any discrepancies between reviewers during the screening process were resolved through discussion and consensus, with the involvement of a third reviewer when necessary.

### 2.2. Eligibility Criteria

Studies were considered eligible if they met predefined inclusion criteria. Specifically, studies were required to have an observational design, including prospective or retrospective cohort studies, and to include adult patients with primary or secondary (functional) MR. Only studies in which patients underwent exercise-based stress echocardiography were included, ensuring methodological consistency across cohorts. Furthermore, eligible studies were required to report clinical outcomes or prognostic endpoints, such as mortality, heart failure, mitral valve intervention, or composite cardiovascular events.

To maintain homogeneity in the assessment of functional response, studies employing exclusively pharmacological stress modalities, such as dobutamine stress echocardiography, were excluded unless an exercise component was clearly incorporated.

Additional exclusion criteria were applied to ensure the relevance and quality of the included evidence. Studies including mixed populations of MR without clearly defined cohorts or without extractable outcome data were excluded. Studies were excluded if they did not provide extractable prognostic data, did not report clinical outcomes, or focused solely on diagnostic performance. Moreover, case reports, editorials, conference abstracts, narrative reviews, and preclinical studies were not considered eligible.

### 2.3. Study Screening and Data Acquisition

Study selection was performed independently by two reviewers. All retrieved records were initially screened based on title and abstract, followed by full-text assessment of potentially eligible studies according to predefined criteria. Disagreements were resolved through consensus, with arbitration by a third reviewer when required.

Data extraction was conducted using a standardized data collection form developed prior to the review. Extracted information included study characteristics, such as first author, year of publication, study design, and sample size, as well as methodological aspects related to the exercise stress protocol.

Clinical and demographic variables were systematically collected when available, including age, sex distribution, cardiovascular risk factors, and comorbid conditions. Information regarding background medical therapy was also recorded when reported.

Echocardiographic and hemodynamic parameters at rest and during exercise were extracted in detail. These included heart rate and blood pressure, along with comprehensive indices of LV structure and function. In particular, LV systolic performance was assessed through LVEF and LV-GLS, while diastolic function and filling pressures were evaluated using Doppler-derived parameters, including the E/e’ ratio.

The severity of MR was characterized using quantitative measures such as EROA and regurgitant volume, as well as categorical grading of MR severity when available. Changes in these parameters during exercise were also recorded to capture the dynamic component of valvular dysfunction.

Pulmonary hemodynamics and RV performance were systematically assessed through sPAP and TAPSE. When available, the TAPSE/sPAP ratio was collected as an index of RV–PA coupling, providing additional insight into ventricular–vascular interaction under stress conditions.

Additional parameters included ventricular volumes, stroke volume, cardiac output, left atrial volume, and indices of functional capacity, such as achieved metabolic equivalents and heart rate recovery.

Clinical outcomes and follow-up data were systematically collected, including duration of follow-up, definition of endpoints, and the main prognostic predictors identified in each study. All extracted data were independently verified by both reviewers, and discrepancies were resolved through re-examination of the original articles.

### 2.4. Evaluation of Methodological Quality and Bias

The methodological quality and risk of bias of the included studies were independently evaluated by two reviewers using the National Institutes of Health (NIH) Quality Assessment Tool for Observational Cohort and Cross-Sectional Studies [20].

This tool assesses multiple methodological domains, including clarity of study objectives, definition of the study population, exposure and outcome assessment, consistency of measurement methods, and appropriateness of statistical analyses. Each domain was rated as “Yes”, “No”, or “Not Reported” according to predefined criteria.

An overall quality judgment was assigned to each study based on the number of criteria fulfilled and the overall methodological rigor. Disagreements between reviewers were resolved through discussion and consensus. The results of the quality assessment were synthesized both descriptively and through graphical representations to provide an overall view of the risk of bias across studies.

### 2.5. Data Integration and Analytical Strategy

Due to the heterogeneity in study design, patient populations, stress protocols, and endpoint definitions, a formal quantitative meta-analysis was not performed. Instead, a structured qualitative and descriptive synthesis of the available evidence was conducted.

To provide an overall characterization of the study population, pooled descriptive estimates were derived from study-level data. Continuous variables were summarized as weighted medians with corresponding interquartile ranges, with weighting based on the sample size of each study. When variables were reported as mean and standard deviation, underlying distributions were approximated assuming normality, allowing harmonization across studies.

This approach enabled consistent summarization of demographic, clinical, and echocardiographic variables across heterogeneous datasets. For parameters reported both at rest and during peak exercise, relative changes between conditions were calculated, providing insight into dynamic physiological responses during stress.

Given the observational nature of the included studies, clinical outcomes and prognostic predictors were synthesized qualitatively rather than quantitatively. Predictors were interpreted within their pathophysiological context and grouped into major domains, including MR severity, ventricular function, pulmonary hemodynamics, clinical variables, biomarkers, and functional capacity. The frequency of reporting across studies was also considered to identify the most consistent and reproducible predictors.

No formal pooling of effect sizes, assessment of heterogeneity, or evaluation of publication bias was performed. However, consistency of findings, directionality of associations, and reproducibility across studies were carefully evaluated to support the robustness of the overall interpretation.

All analyses were conducted at the study level. Data processing and aggregation were performed using standard spreadsheet software (Microsoft Excel, Microsoft Corporation, Redmond, WA, USA).

### 2.6. Artificial Intelligence-Assisted Language Refinement

Artificial intelligence tools were used exclusively to support language refinement during manuscript preparation. ChatGPT-5.3 (OpenAI, San Francisco, CA, USA) was employed to improve grammar, clarity, and readability. No AI-based tools were used for study design, data collection, statistical analysis, or interpretation of results. All scientific content and conclusions were independently developed and verified by the authors.

## 3. Results

### 3.1. Literature Search and Study Selection

The systematic search across PubMed, Scopus, and EMBASE databases yielded a total of 277 records. After removal of duplicate entries (n = 17), 260 unique studies were considered for screening. Title and abstract evaluation led to the exclusion of 230 articles that did not meet the predefined inclusion criteria.

Subsequently, 30 studies were retrieved for full-text assessment. Among these, 11 were excluded due to the lack of extractable prognostic data relevant to the study objectives.

**Figure 1:**
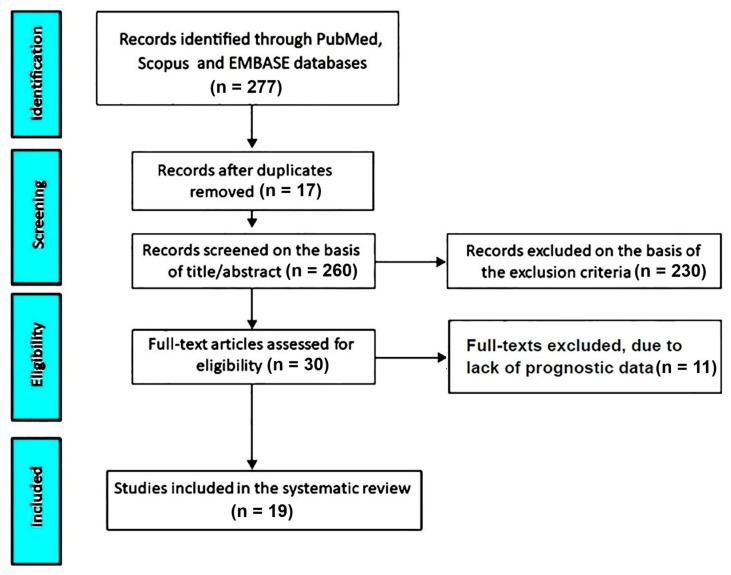
PRISMA flow diagram of study screening and selection.

As a result, 19 studies [21,22,23,24,25,26,27,28,29,30,31,32,33,34,35,36,37,38,39] fulfilled all eligibility criteria and were included in the final qualitative synthesis. The study selection workflow is illustrated in Figure 1.

### 3.2. Characteristics of the Included Studies

The key features of the studies included in the present analysis are summarized in Table 1.

Overall, 19 studies published between 2003 and 2025 were considered, encompassing both prospective and retrospective observational designs. Most investigations were conducted in single-center settings, although a subset of multicenter studies was also represented. Despite differences in design, the methodological approaches were broadly consistent across studies, particularly regarding stress echocardiography protocols and patient selection criteria.

Exercise stress echocardiography was predominantly performed using a semi-supine bicycle protocol with stepwise incremental workloads, typically starting between 10 and 30 W and increasing at regular intervals of 2–3 min. Treadmill-based stress testing, mainly following standard or modified Bruce protocols, was adopted in several studies, while one investigation integrated cardiopulmonary exercise testing with echocardiographic assessment. Overall, the stress modalities were applied in a symptom-limited fashion, reflecting real-world clinical practice.

The study populations were heterogeneous but largely centered on patients with primary or secondary MR undergoing functional evaluation. Several cohorts included asymptomatic or mildly symptomatic individuals with preserved LVEF, whereas others focused on patients with LV dysfunction or heart failure with reduced ejection fraction. Specific subgroups, such as exercise-induced MR or ischemic MR candidates for surgical intervention, were also represented.

Sample sizes varied widely across studies, ranging from small cohorts of fewer than 50 patients to large observational series including several hundred participants. Across the included studies, the proportion of male patients was consistently high, generally exceeding 60%, indicating a predominance of male subjects within the analyzed populations.

Taken together, the included studies provide a comprehensive overview of different clinical scenarios of MR, encompassing a broad spectrum of disease severity and underlying pathophysiological mechanisms. This diversity enhances the generalizability of the findings while maintaining sufficient methodological consistency to support the comparative evaluation of stress echocardiography-derived prognostic markers.

### 3.3. Baseline Patient Profile

Baseline demographic and clinical characteristics of the pooled population are detailed in Table 2.

The analysis included 5555 patients, who were predominantly middle-aged to elderly, with a weighted median age of 58.5 years. A marked male predominance was observed, with men accounting for approximately two-thirds of the study population.

Anthropometric data, available in a subset of studies, indicated a generally overweight profile, with a median body mass index of 25.7 kg/m^2^ and relatively consistent body surface area values across cohorts. However, reporting of these parameters was not uniform, reflecting heterogeneity in data availability among studies.

Cardiovascular risk factors were frequently reported. Hypertension and dyslipidemia represented the most prevalent comorbidities, whereas diabetes mellitus and prior cerebrovascular events were less common but consistently documented. A history of smoking was also present in a considerable proportion of patients, supporting the presence of a moderate cardiovascular risk profile. Coronary artery disease and atrial fibrillation were variably represented across studies, while chronic kidney disease was only sporadically reported.

Medical therapy was largely consistent with contemporary standards of cardiovascular care. Renin–angiotensin system inhibitors and beta-blockers were widely prescribed, whereas antiplatelet therapy was reported in a relevant proportion of patients. Other treatments, including diuretics, mineralocorticoid receptor antagonists, calcium channel blockers, and nitrates, showed greater variability, likely reflecting differences in clinical status and reporting practices.

Biomarker data were available in a limited number of studies. Circulating natriuretic peptides, including B-type natriuretic peptide (BNP) and N-terminal pro–B-type natriuretic peptide (NT-proBNP), were reported in selected cohorts, with median values within a low-to-moderate range. Similarly, surgical risk assessment using the Society of Thoracic Surgeons (STS) score was documented in only a few studies, suggesting a generally low operative risk among included patients.

Collectively, the pooled cohort reflects a clinically representative spectrum of patients with MR, characterized by a moderate burden of cardiovascular comorbidities and heterogeneous clinical presentations. This variability provides a solid framework for the evaluation of prognostic markers derived from stress echocardiography.

### 3.4. Resting and Exercise Echocardiographic Findings

Table 3 summarizes echocardiographic and hemodynamic parameters at rest and during exercise.

At baseline, patients showed preserved or mildly reduced LV systolic function, with median LVEF values around 58%, alongside ventricular dimensions and volumes consistent with the heterogeneous etiologies of MR. Resting measurements indicated a compensated hemodynamic profile, with relatively stable stroke volume and cardiac output, and no clear evidence of advanced decompensation in most cases.

During exercise, a pronounced physiological response was observed. Heart rate and systolic blood pressure increased substantially, confirming an appropriate chronotropic and pressor adaptation to stress. This was paralleled by a rise in cardiac output, reflecting the expected augmentation of forward flow under exercise conditions.

Importantly, several disease-specific parameters exhibited significant dynamic changes. Both EROA and regurgitant volume increased during exercise, highlighting the dynamic nature of MR severity. Consistently, the proportion of patients with moderate-to-severe regurgitation rose under stress conditions, supporting the concept of exercise-induced worsening of valvular incompetence.

In parallel, indices of LV filling pressure increased, as reflected by higher E/e’ values during exercise, suggesting a limited diastolic reserve. This was accompanied by a marked rise in pulmonary artery pressures, indicating transmission of elevated left-sided pressures to the pulmonary circulation. Despite these changes, longitudinal systolic function assessed by GLS showed only modest variation, suggesting preserved contractile reserve in a subset of patients.

Right ventricular function demonstrated a mixed response. Although TAPSE increased during exercise, indicating enhanced longitudinal shortening, the TAPSE/sPAP ratio decreased, suggesting impaired RV–PA coupling under stress conditions.

Of note, the type of exercise modality influenced the extent and granularity of echocardiographic data acquisition. Studies employing semi-supine bicycle ergometry more consistently reported paired rest–exercise (Δ) measurements across a broad range of parameters, including EROA, regurgitant volume, E/e’, and pulmonary pressures. This likely reflects the feasibility of continuous image acquisition during exercise. In contrast, treadmill-based studies more frequently relied on post-exercise imaging, resulting in a more limited and less systematic assessment of dynamic changes. This methodological difference may have contributed to variability in the completeness and comparability of stress-derived parameters across studies.

### 3.5. Clinical Outcomes and Predictors of Prognosis

Table 4 presents clinical outcomes and their main predictors across the included studies.

Follow-up duration varied substantially, ranging from approximately 13 to nearly 100 months. Event rates were heterogeneous across studies, spanning from about 9% to over 40%, reflecting differences in patient characteristics, disease severity, and endpoint definitions.

The most commonly reported outcomes included composite cardiovascular events, progression to heart failure, need for mitral valve intervention, and mortality. Despite variability in endpoint definitions, the overall burden of adverse events was clinically meaningful, underscoring the importance of early risk stratification even in patients with limited or absent symptoms.

A wide range of prognostic markers emerged from ESE. Parameters reflecting the dynamic severity of MR played a central role, with both resting and exercise-derived EROA consistently associated with adverse outcomes. In particular, exercise-induced worsening of regurgitation and the development of severe MR under stress conditions were among the most robust predictors.

Indices of LV function were also highly relevant. The absence of contractile reserve, whether assessed by LVEF or GLS, was repeatedly associated with poorer prognosis. Similarly, impaired systolic performance during exercise and reduced longitudinal deformation emerged as important markers of subclinical ventricular dysfunction.

Hemodynamic parameters obtained during stress provided additional prognostic information. Elevated filling pressures, as reflected by increased E/e’ during exercise, and the development of exercise-induced pulmonary hypertension were consistently linked to adverse outcomes. Furthermore, indices of RV–PA coupling, such as TAPSE/sPAP ratio, demonstrated prognostic value, highlighting the importance of ventricular–vascular interaction in this setting.

Beyond echocardiographic parameters, clinical and functional variables also contributed to risk stratification. Age, atrial fibrillation, diabetes mellitus, and reduced exercise capacity [including lower achieved metabolic equivalents (METs) and impaired heart rate recovery] were frequently identified as significant predictors. Biomarkers, particularly natriuretic peptides, as well as surgical risk scores such as STS, provided complementary prognostic information.

**Figure 2:**
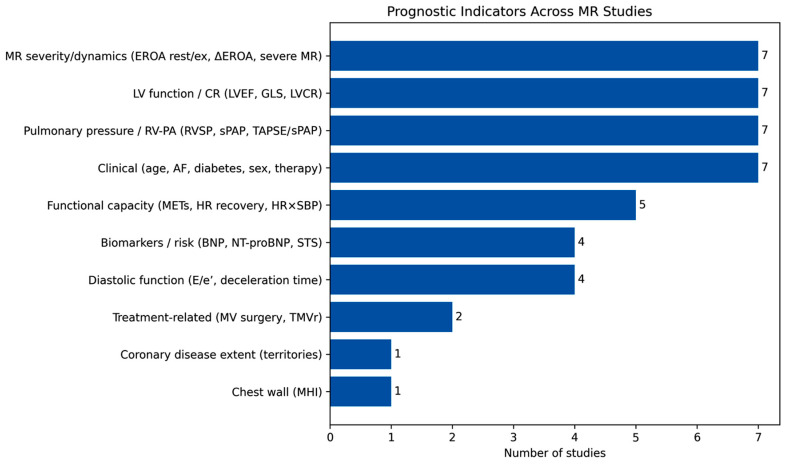
Distribution of Prognostic Marker Categories Across Included Studies. AF, atrial fibrillation; BNP, brain natriuretic peptide; CR, contractile reserve; E/e’, ratio of early transmitral flow velocity to early diastolic mitral annular velocity; EROA, effective regurgitant orifice area; GLS, global longitudinal strain; HR, heart rate; LVCR, left ventricular contractile reserve; LVEF, left ventricular ejection fraction; METs, metabolic equivalents; MHI, modified Haller index; MR, mitral regurgitation; NT-proBNP, N-terminal pro–brain natriuretic peptide; RV, right ventricle; RVSP, right ventricular systolic pressure; sPAP, systolic pulmonary artery pressure; STS, Society of Thoracic Surgeons; TAPSE, tricuspid annular plane systolic excursion; TMVr, transcatheter mitral valve repair.

**Figure 3:**
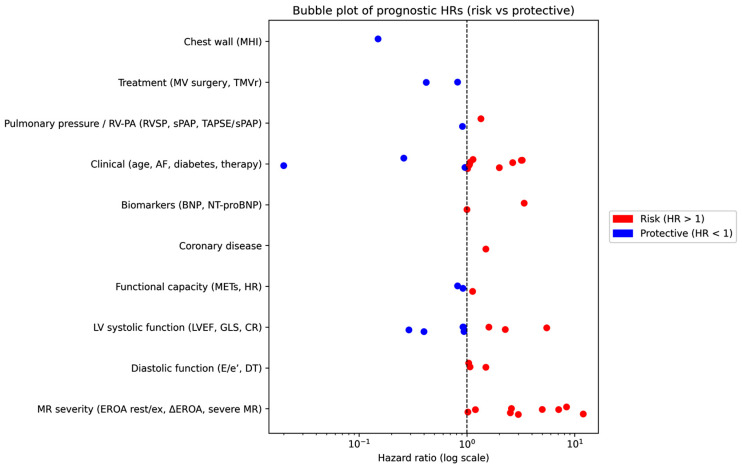
Bubble Plot of Hazard Ratios for Risk and Protective Predictors. Each point represents an individual estimate, plotted on a logarithmic scale, with the vertical reference line indicating a hazard ratio of 1. Values to the right of the reference line denote variables associated with higher risk, whereas those to the left indicate protective associations. AF, atrial fibrillation; BNP, brain natriuretic peptide; CR, contractile reserve; DT, deceleration time; E/e’, ratio of early transmitral flow velocity to early diastolic mitral annular velocity; EROA, effective regurgitant orifice area; GLS, global longitudinal strain; HR, hazard ratio; LVEF, left ventricular ejection fraction; METs, metabolic equivalents; MHI, modified Haller index; MR, mitral regurgitation; NT-proBNP, N-terminal pro–brain natriuretic peptide; RV, right ventricle; RVSP, right ventricular systolic pressure; sPAP, systolic pulmonary artery pressure; TAPSE, tricuspid annular plane systolic excursion; TMVr, transcatheter mitral valve repair.

These findings are visually synthesized in Figure 2 and Figure 3, which provide a complementary graphical representation of the prognostic landscape derived from the included studies.

Figure 2 provides a structured overview of the frequency with which different categories of prognostic markers were reported across studies. Variables related to MR severity/dynamics, LV function, pulmonary pressures, and clinical characteristics were the most consistently investigated domains. Functional capacity indices, biomarkers, and diastolic parameters were also commonly reported, whereas treatment-related factors and less frequently assessed variables appeared in a limited number of studies. This distribution further supports the multidimensional nature of risk stratification in MR, integrating structural, functional, and clinical determinants.

Figure 3 illustrates the distribution of individual hazard ratios across predefined predictor domains, distinguishing between risk-associated (HR > 1) and protective (HR < 1) factors. Notably, parameters related to MR severity and dynamics, as well as indices of LV systolic function, clustered predominantly in the higher-risk range, with several markers showing markedly elevated hazard ratios. In contrast, selected functional and treatment-related variables exhibited protective associations, reflecting the heterogeneity of prognostic profiles across studies.

### 3.6. Risk of Bias and Study Quality Evaluation

**Figure 4:**
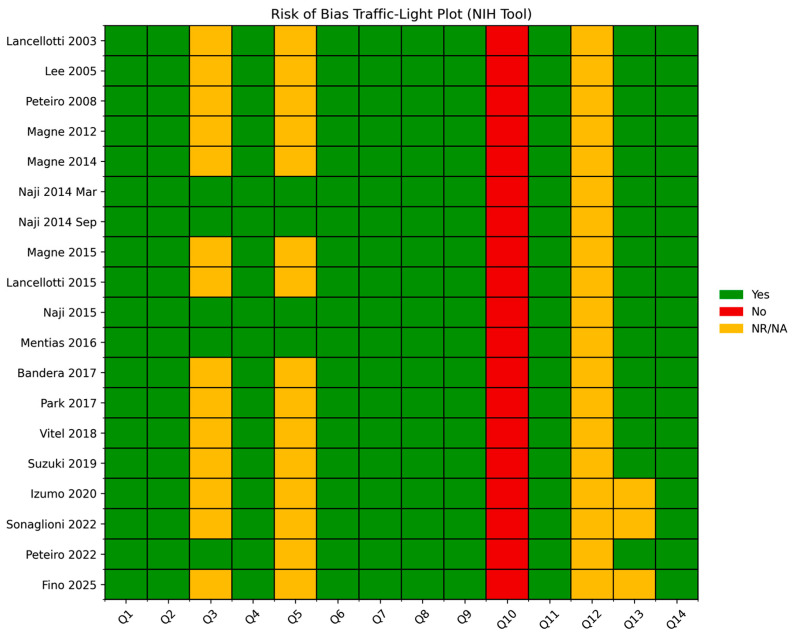
Risk of bias traffic-light plot across studies [21,22,23,24,25,26,27,28,29,30,31,32,33,34,35,36,37,38,39] according to the NIH Quality Assessment Tool. Each row represents an individual study, while each column corresponds to a specific methodological domain (Q1–Q14). Color coding indicates the level of bias risk: green denotes low risk (“Yes”), red indicates high risk (“No”), and yellow represents insufficient or unclear reporting (“Not Reported/Not Applicable”). Overall, most domains demonstrated a low risk of bias, with only a limited number of criteria showing incomplete reporting or potential methodological concerns. NIH, National Institutes of Health; NR/NA, not reported/not applicable; Q, question.

**Figure 5:**
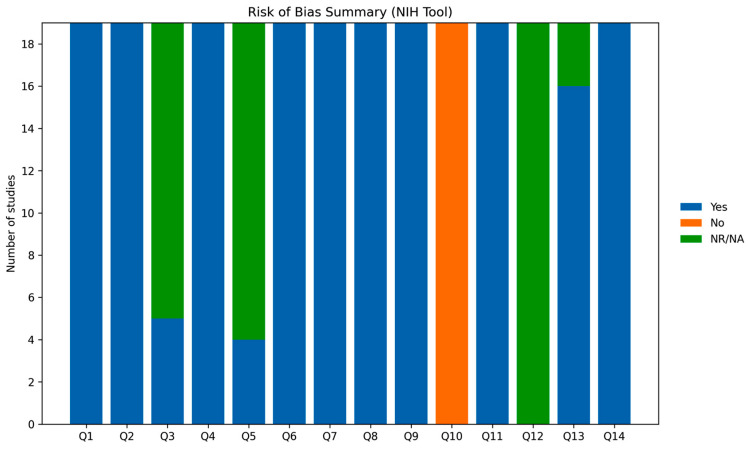
Summary of risk of bias across studies [21,22,23,24,25,26,27,28,29,30,31,32,33,34,35,36,37,38,39] according to the NIH Tool. Each bar represents the distribution of ratings for a specific domain (Q1–Q14), expressed as the number of studies fulfilling each criterion. Most domains demonstrated a high proportion of “Yes” ratings, indicating overall good methodological quality. However, selected domains—particularly those related to sample size justification and reporting completeness—showed a higher proportion of “Not Reported/Not Applicable” or “No” ratings, reflecting areas of potential methodological limitation. NIH, National Institutes of Health; NR/NA, not reported/not applicable; Q, question.

The methodological rigor of the included studies [21,22,23,24,25,26,27,28,29,30,31,32,33,34,35,36,37,38,39] is reported in Table 5, with visual summaries provided in Figure 4 and Figure 5.

Based on the NIH Quality Assessment Tool, all studies were classified as having an generally acceptable methodological quality with several recurring limitations. Across domains, most criteria were consistently fulfilled, particularly those related to the clarity of study objectives, definition of the study population, and assessment of exposures and outcomes. These elements indicate a moderate risk of bias overall, with relatively robust performance in key methodological domains rather than uniformly low risk.

Nevertheless, some recurrent limitations were identified. In particular, justification of sample size and reporting of power calculations were frequently lacking. In addition, information regarding blinding of outcome assessors was often absent or insufficiently detailed. Furthermore, heterogeneity in study design and variability in outcome definitions across studies may have contributed to additional sources of bias and limit comparability. These gaps are reflected in the graphical representations, where selected domains show a proportion of “not reported” responses, along with occasional instances classified as “no”.

Despite these limitations, the overall methodological profile was reasonably consistent across studies, although not without important weaknesses, supporting the internal consistency of the evidence while warranting cautious interpretation of the findings.

## 4. Discussion

### 4.1. Principal Findings

This systematic review provides a comprehensive overview of the prognostic role of ESE in patients with MR, highlighting the limitations of relying exclusively on resting assessment and symptom status for risk stratification.

Across the included studies, a considerable proportion of patients classified as asymptomatic or mildly symptomatic experienced adverse clinical events during follow-up. This observation reinforces the concept that clinical stability in MR may be misleading, as progressive hemodynamic burden and ventricular remodeling can occur silently over time. Consequently, symptom-based evaluation alone may fail to capture the true severity and evolution of the disease.

In this context, ESE emerges as a valuable tool for uncovering dynamic abnormalities that remain unapparent at rest. One of the most consistent findings across studies is the prognostic relevance of exercise-induced changes in MR severity. In particular, increases in EROA and regurgitant volume during stress reflect the load-dependent nature of MR and identify patients with limited adaptive reserve and higher risk of adverse outcomes.

In addition, indices of LV function assessed during exercise provided important prognostic information. The absence of contractile reserve, evaluated through changes in LVEF or GLS, was consistently associated with worse outcomes. Similarly, exercise-induced elevation of LV filling pressures, as reflected by E/e’, further contributed to risk stratification, indicating impaired diastolic reserve.

Hemodynamic responses involving the pulmonary circulation also emerged as key determinants of prognosis. The development of exercise-induced pulmonary hypertension and alterations in RV–PA coupling, particularly reduced TAPSE/sPAP ratio, highlighted the impact of MR on the cardiopulmonary unit and its relevance in identifying advanced disease stages.

Overall, the prognostic value of ESE appears to derive from its ability to provide a multidimensional assessment of MR, integrating valvular severity, ventricular performance, and hemodynamic adaptation to stress. This comprehensive approach offers a more accurate representation of disease burden compared with resting evaluation alone and helps explain why stress-derived parameters consistently demonstrate strong associations with clinical outcomes.

### 4.2. Pathophysiological Basis of Stress-Induced Abnormalities in Mitral Regurgitation

The pathophysiological mechanisms underlying the prognostic relevance of exercise-derived parameters in MR are rooted in the dynamic nature of the disease and its complex interaction with ventricular and atrial remodeling [40]. Unlike fixed valvular lesions, MR is highly load-dependent, and its severity may substantially change during exercise, reflecting the interplay between preload, afterload, and LV contractile function [41].

Under resting conditions, compensatory mechanisms—primarily LV dilation and increased compliance—maintain forward stroke volume despite significant regurgitant flow [42,43]. This adaptive phase often preserves LVEF, potentially masking early myocardial dysfunction; however, these reserves may be unmasked during exercise, when the hemodynamic burden increases and latent abnormalities become evident [44].

A key mechanism is the dynamic increase in MR severity during stress. Exercise-induced enlargement of the EROA and regurgitant volume reflects worsening leaflet coaptation and increased tethering forces in functional MR, or enhanced prolapse dynamics in degenerative disease [45]. This phenomenon has been consistently associated with adverse outcomes, identifying patients with limited hemodynamic reserve and higher risk of disease progression [46,47].

At the myocardial level, impaired contractile reserve represents an early marker of dysfunction. The inability to augment LVEF or GLS during exercise reflects subclinical LV impairment, even in the presence of preserved resting systolic function [48]. GLS-based assessment appears particularly sensitive for detecting early myocardial damage and has been independently associated with worse outcomes [49,50].

Diastolic dysfunction further contributes to exercise intolerance and prognosis. Exercise-induced elevation of LV filling pressures, commonly assessed by E/e’, reflects reduced diastolic reserve and increased chamber stiffness, often preceding overt abnormalities at rest [51,52,53]. These changes are indicative of early myocardial remodeling and fibrosis and promote a rise in left atrial pressure, which is transmitted to the pulmonary circulation [54,55].

Consequently, pulmonary vascular involvement and RV function become integral components of disease progression. Exercise-induced increases in sPAP are consistently associated with adverse outcomes, while impaired RV–PA coupling—particularly reduced TAPSE/sPAP ratio—identifies patients with compromised cardiopulmonary interaction and reduced functional capacity [56,57,58,59].

Collectively, these findings support a pathophysiological model in which MR represents a dynamic and systemic condition rather than an isolated valvular lesion. The progressive involvement of the left ventricle, left atrium, pulmonary circulation, and right ventricle reflects a continuum of cardiac damage. In this context, ESE enables early identification of patients transitioning from a compensated to a decompensated state, providing mechanistic insight into the prognostic significance of exercise-derived parameters.

### 4.3. Clinical Implications

The findings of the present analysis have important implications for the clinical management of patients with primary or secondary MR, although the greatest impact of ESE may be in asymptomatic or minimally symptomatic individuals with discordant clinical and echocardiographic profiles. In this setting, decision-making remains challenging, as current strategies still rely largely on resting measurements and the onset of symptoms, which may occur late in the disease course.

According to the most recent European Society of Cardiology and European Association for Cardio-Thoracic Surgery guidelines [60], indications for intervention are primarily driven by symptom status, LV systolic dysfunction, or evidence of cardiac remodeling, along with selected additional criteria such as atrial fibrillation or pulmonary hypertension. However, these recommendations are predominantly based on resting parameters and may not fully capture the dynamic nature of the disease or identify patients with early functional impairment.

In this context, the evidence synthesized in the present review underscores the incremental value of a dynamic, stress-based evaluation. ESE provides a more comprehensive assessment of MR severity, ventricular performance, and cardiopulmonary interaction under physiological conditions that more closely reflect daily life. This approach enables the detection of latent abnormalities not evident at rest, thereby improving risk stratification and offering a more accurate representation of disease burden.

Across the included studies, several exercise-derived parameters consistently emerged as markers of adverse outcome, including exercise-induced worsening of MR, impaired contractile reserve, elevated filling pressures, and the development of pulmonary hypertension. Although standardized thresholds remain to be definitively established, these findings collectively support the role of ESE as a key tool for identifying patients at higher risk and for complementing current guideline-based criteria.

Overall, these observations support a shift from a purely static, rest-based assessment toward a more dynamic and integrative model of evaluation, in which functional reserve and exercise-induced changes provide clinically meaningful information beyond conventional measurements.

However, it should be emphasized that the available evidence is largely derived from observational studies, and therefore primarily supports risk stratification rather than direct clinical benefit. In particular, randomized controlled trials demonstrating that ESE-guided management strategies lead to improved clinical outcomes are currently lacking. Consequently, while ESE may refine risk assessment and inform clinical decision-making, its impact on patient prognosis when used to guide therapeutic interventions remains to be definitively established.

### 4.4. Emerging and Non-Conventional Prognostic Indicators

Beyond traditional echocardiographic and stress-derived parameters, growing evidence suggests that extracardiac anatomical features—particularly chest wall configuration—may significantly influence the clinical expression and prognosis of primary MR. In this context, the Modified Haller Index (MHI), which reflects the relationship between the transverse thoracic diameter and the antero-posterior (A–P) distance between sternum and spine, provides a simple and non-invasive descriptor of thoracic geometry [61].

Recent studies have highlighted how thoracic conformation is not merely an anatomical variant, but may be closely associated with distinct cardiac phenotypes. Specifically, a reduced A–P thoracic diameter—typically ≤13.5 cm and corresponding to a “narrow” or concave chest (MHI ≥ 2.5)—is frequently associated with smaller left atrial dimensions, mitral valve prolapse, and forms of mitral annular disjunction that are often benign [62]. In these patients, alterations in myocardial deformation parameters may be observed; however, such findings are more likely related to extrinsic cardiac compression and geometric displacement rather than true intrinsic myocardial dysfunction [63]. Consistently, this phenotype has been associated with a more favorable clinical course, even in the presence of moderate MR, with a lower incidence of adverse cardiovascular events during follow-up [37].

In contrast, patients with a larger A–P thoracic diameter—typically ≥18 cm and corresponding to a “wide” chest configuration (MHI < 2.5)—tend to exhibit a different structural and functional profile. In this setting, the left atrium and mitral annulus are more frequently dilated, and MR is more likely to be significant and hemodynamically relevant [64]. Unlike the narrow chest phenotype, where apparent dysfunction may be largely geometric, these patients appear more prone to true myocardial involvement, which may translate into a higher-risk clinical trajectory.

**Figure 6:**
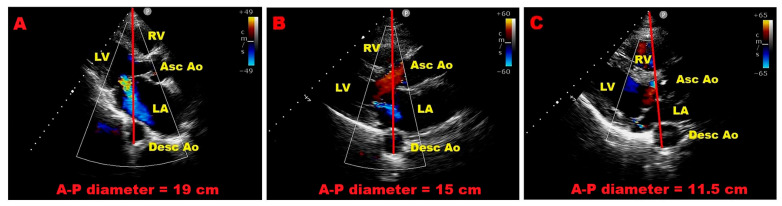
Representative transthoracic echocardiographic images illustrating the relationship between thoracic geometry and mitral regurgitation (MR) severity. Panel (**A**) shows a patient with a wide chest configuration (antero-posterior [A–P] diameter = 19 cm), associated with severe MR. Panel (**B**) depicts a patient with a normal thoracic configuration (A–P diameter = 15 cm) and moderate MR. Panel (**C**) illustrates a patient with a narrow chest configuration (A–P diameter = 11.5 cm), associated with mild MR. The red line represents the antero-posterior thoracic diameter, measured as the distance between the ultrasound entry point on the chest wall and the posterior wall of the descending aorta, immediately behind the left atrium. LV = left ventricle; LA = left atrium; RV = right ventricle; Asc Ao = ascending aorta; Desc Ao = descending aorta.

As an illustrative example of these concepts, Figure 6 shows representative echocardiographic images of patients with different thoracic configurations and corresponding severity of MR. A wide chest phenotype is associated with more severe MR, whereas a narrow chest configuration is typically observed in patients with milder forms of regurgitation.

Taken together, these observations support the concept that chest wall morphology may act as an important modifier of disease expression in primary MR. A narrow chest configuration seems to identify a subgroup characterized by mechanical constraint, smaller cardiac chambers, and generally benign forms of mitral valve disease, whereas a wide chest configuration is more often associated with structural remodeling, annular dilation, and clinically significant regurgitation. Although these findings remain exploratory and require validation in larger cohorts, integrating thoracic geometry into routine assessment may help refine risk stratification, particularly in patients with discordant imaging findings or borderline indications for intervention.

Importantly, the evidence supporting the routine clinical implementation of chest wall morphology assessment, including the MHI, remains preliminary, and its use should currently be considered exploratory rather than established in standard clinical practice.

### 4.5. Integrating Stress-Derived and Anatomical Parameters for Risk Stratification in Mitral Regurgitation

Based on the integration of stress-derived functional parameters and chest wall morphology, a practical and clinically oriented approach for risk stratification in patients with MR can be proposed.

In patients without clear guideline-based indications for intervention, ESE should be performed to assess dynamic changes in MR severity, LV functional reserve, and cardiopulmonary interaction. Particular attention should be given to predefined high-risk features, including an exercise-induced increase in EROA ≥ 0.13 cm^2^, lack of contractile reserve (defined as a LVEF increase <5% or impaired augmentation of GLS), elevated LV filling pressures (peak E/e’ ≥ 15), and the development of exercise-induced pulmonary hypertension (peak sPAP ≥ 60 mmHg). It is worth noting that these threshold values are derived from observational studies and heterogeneous populations, and therefore should be interpreted with caution rather than considered as definitive or guideline-endorsed cut-offs.

These functional findings can be considered alongside anatomical features, including chest wall configuration. A reduced A–P thoracic diameter (≤13.5 cm), corresponding to an MHI ≥ 2.5, identifies a “narrow chest” phenotype, often associated with smaller cardiac chambers and potentially more favorable hemodynamic adaptation. In contrast, an increased A–P diameter (≥18 cm), reflecting a “wide chest” configuration (MHI < 2.5), is more frequently associated with left atrial enlargement, mitral annular dilation, and more severe regurgitation patterns.

**Figure 7:**
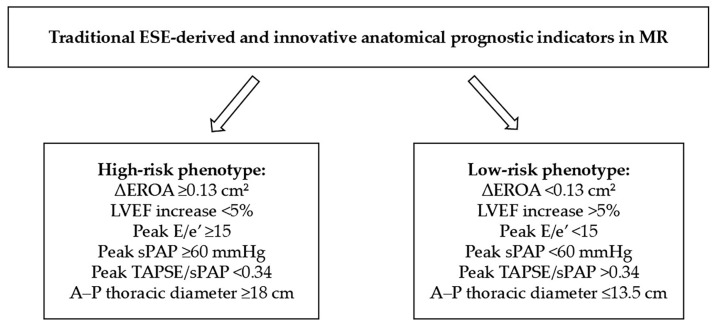
Schematic representation of a stepwise approach integrating ESE-derived functional parameters and anatomical features for risk stratification in patients with MR. A–P, antero-posterior; ΔEROA, change in effective regurgitant orifice area; E/e’, ratio of early mitral inflow velocity to mitral annular early diastolic velocity; EROA, effective regurgitant orifice area; ESE, exercise stress echocardiography; LVEF, left ventricular ejection fraction; MHI, Modified Haller Index; MR, mitral regurgitation; sPAP, systolic pulmonary artery pressure; TAPSE, tricuspid annular plane systolic excursion.

The combined interpretation of stress-derived and anatomical parameters allows classification of patients into two clinically relevant phenotypes, reflecting opposite ends of the risk spectrum. A high-risk phenotype is defined by the presence of one or more abnormal stress-derived parameters—particularly exercise-induced MR progression, impaired contractile reserve, elevated E/e’, or pulmonary hypertension—especially when associated with adverse structural features (typically wide chest configuration). In this subgroup, early referral to a multidisciplinary Heart Team and consideration of intervention may be appropriate, even in the absence of conventional indications. In contrast, a low-risk phenotype is characterized by the absence of exercise-induced worsening of MR, preserved contractile reserve, normal LV filling pressures, and a favorable anatomical profile (typically narrow chest configuration); these patients may be managed conservatively with periodic follow-up (Figure 7).

This integrated approach moves beyond a purely parameter-based evaluation toward a phenotype-oriented model, in which functional reserve and anatomical context are jointly considered. Such a strategy may improve the identification of patients at risk of disease progression across the spectrum of MR and support more timely and individualized therapeutic decisions.

### 4.6. Sources of Variability Across Studies, Strengths and Limitations

The interpretation of the present findings should take into account several sources of variability across the included studies, which may have influenced both the magnitude of observed effects and the consistency of prognostic results.

Heterogeneity in study design represents an important factor, as the inclusion of both prospective and retrospective cohorts, often characterized by different enrollment strategies and referral pathways, may have introduced selection bias and affected the clinical profile of the populations as well as the reported incidence of adverse events.

In addition, patient populations were inherently heterogeneous, encompassing a broad spectrum of MR phenotypes, including both primary and secondary forms with varying severity and distinct pathophysiological mechanisms, ranging from leaflet degeneration to ventricular remodeling. Importantly, primary (degenerative) and secondary (functional) MR represent fundamentally different disease entities, with divergent natural history, response to intervention, and prognostic determinants. Consequently, pooling and qualitatively integrating findings across these groups may reduce clinical specificity, and the prognostic implications of exercise-derived parameters may not be directly transferable. Therefore, the results of the present review should be interpreted with caution, as they may not apply equally to primary and secondary MR.

Further variability arises from differences in exercise protocols, including the use of semi-supine bicycle versus treadmill testing, as well as variations in workload increments, test duration, and termination criteria, all of which may have affected hemodynamic responses and the detection of dynamic changes in MR severity.

Methodological differences in echocardiographic acquisition and analysis also contribute to inter-study variability, given the operator dependency of measurements and potential inconsistencies in the assessment of parameters such as EROA, regurgitant volume, pulmonary pressures, and myocardial deformation indices. Moreover, differences in the timing of image acquisition, whether at peak exercise or during early recovery, may have influenced reported values.

Finally, heterogeneity in outcome definitions and follow-up duration, with endpoints ranging from composite cardiovascular events to mortality or intervention-driven outcomes, further limits direct comparability across studies.

Despite these sources of variability, this systematic review provides a structured and comprehensive synthesis of the available evidence on the prognostic role of ESE in MR. The adoption of a standardized methodological approach, including PRISMA-guided selection and formal quality assessment, represents a major strength, allowing integration of findings across a broad spectrum of clinical settings.

Nevertheless, some limitations should be acknowledged. The analysis is based on study-level data, precluding detailed adjustment for confounding factors and limiting the exploration of interactions between clinical and echocardiographic variables. The absence of a formal meta-analysis, due to substantial heterogeneity in study design, populations, and endpoints, prevents quantitative estimation of pooled effect sizes. In addition, incomplete reporting of certain variables across studies may have reduced the robustness of comparative analyses, and publication bias cannot be excluded.

From a methodological perspective, ESE itself presents intrinsic limitations, including technical challenges in image acquisition during exercise, operator dependency, and variability related to the load-dependent nature of MR, which may affect the reproducibility and accuracy of measurements [65,66,67,68,69,70].

Furthermore, several practical barriers may limit the widespread implementation of ESE in routine clinical practice, including the need for specific operator expertise and training, time constraints during examination, resource and equipment availability, and patient-related factors such as limited exercise capacity or suboptimal tolerance to stress protocols [11,71].

These considerations highlight the need for larger, standardized, and methodologically rigorous studies to further define the role of ESE in the evaluation and management of MR.

### 4.7. Future Perspectives

Future research should focus on further clarifying the role of ESE in the management of patients with MR, particularly in those with asymptomatic or minimally symptomatic disease, where clinical decision-making remains challenging.

Large prospective, multicenter studies are needed to validate the prognostic impact of exercise-derived parameters and to define robust and reproducible thresholds that can be translated into clinical practice. In particular, the identification of standardized cut-off values for dynamic changes in MR severity, indices of contractile reserve, and markers of elevated LV filling pressures during exercise represents a key step toward improving risk stratification.

In addition, the incorporation of advanced imaging techniques may enhance the sensitivity of ESE in detecting early myocardial dysfunction. Parameters such as LV-GLS and RV–PA coupling have shown promising prognostic value and may provide incremental information beyond conventional measures. The integration of ESE with cardiopulmonary exercise testing and circulating biomarkers could further refine the evaluation of functional capacity and hemodynamic reserve, allowing a more comprehensive characterization of disease burden [72].

Furthermore, future research should explore the systematic integration of extracardiac anatomical features into the clinical assessment of MR. In particular, chest wall configuration, as quantified by the MHI, may represent a simple and reproducible parameter capable of refining risk stratification by identifying distinct phenotypic subsets of patients. The implementation of MHI assessment in routine clinical practice, in combination with stress-derived functional parameters, could contribute to a more individualized and pathophysiology-oriented approach to patient management. However, dedicated prospective studies are required to validate its clinical utility and to define its role within integrated diagnostic algorithms.

Technological developments, including artificial intelligence and machine learning approaches, may facilitate the integration of multiparametric data and support the identification of complex risk profiles that are not easily captured by traditional analytical methods.

Future investigations should aim to incorporate ESE-derived variables into structured clinical pathways and to evaluate, through randomized studies, whether management strategies guided by stress-induced abnormalities can improve timing of intervention and long-term outcomes in patients with MR.

From a health system perspective, future research should address the cost-effectiveness of ESE-based strategies in the context of valvular surveillance and stress imaging. As healthcare systems face increasing resource constraints and a growing burden of valvular heart disease, understanding the economic impact and sustainability of advanced imaging approaches will be essential to support their broader clinical implementation and integration into routine care pathways [73].

## 5. Conclusions

Exercise stress echocardiography provides relevant incremental prognostic information in patients with MR by uncovering dynamic abnormalities that are not apparent during resting evaluation. The ability to assess changes in regurgitation severity, ventricular function, filling pressures, and cardiopulmonary interaction under physiological stress enables a more comprehensive characterization of disease burden.

The available evidence consistently demonstrates that exercise-induced parameters—particularly those reflecting worsening regurgitation, impaired contractile reserve, and elevated filling or pulmonary pressures—are closely associated with adverse clinical outcomes. These findings highlight the added value of stress-based assessment over conventional resting measurements for risk stratification.

Incorporating exercise-derived information into routine clinical evaluation may facilitate a more individualized management strategy, allowing earlier identification of patients at risk of disease progression and potentially improving the timing of intervention.

Further well-designed prospective studies are needed to standardize acquisition protocols, validate reproducible thresholds, and clarify the clinical impact of ESE-guided decision-making in patients with MR.

## Figures and Tables

**Table 1:** Characteristics of Included Studies [21,22,23,24,25,26,27,28,29,30,31,32,33,34,35,36,37,38,39] and Exercise Stress Echocardiography Protocols.

Study Name, Year and Country	Design	Stress Modality	Workload Protocol	Population	Size (% Males)
Lancellotti P. (2003), Belgium [21]	Prospective, single-center	Semi-supine bicycle ergometer	25 W for 6 min, then +25 W every 2 min	Ischemic LV dysfunction (LVEF ≤ 45%) with ≥ mild MR	98 (67%)
Lee R. (2005), Australia [22]	Prospective, single-center	Treadmill or upright bicycle	Bruce protocol or stepwise bicycle (+25 W every 2 min)	Asymptomatic severe primary (degenerative) MR, NYHA I–II, preserved LVEF	71 (68%)
Peteiro J. (2008), Spain [23]	Retrospective, single-center	Treadmill exercise echocardiography	Bruce (86%), modified Bruce (11%), Naughton (3%); symptom-limited	Functional MR with LVEF ≤ 45%	323 (81%)
Magne J. (2012), Belgium/Canada [24]	Prospective, multicenter	Semi-supine bicycle ergometer	25 W start, +25 W every 2 min	Asymptomatic moderate-to-severe primary MR, LVEF > 60%	113 (59%)
Magne J. (2014), Belgium/Canada [25]	Prospective, multicenter	Semi-supine bicycle ergometer	25 W start, +25 W every 2 min	Asymptomatic moderate-to-severe primary MR, LVEF > 60%	115 (56%)
Naji P. (2014), USA [26]	Retrospective, single-center	Treadmill exercise echocardiography	Standard Bruce protocol	Asymptomatic or mildly symptomatic ≥ grade III myxomatous MR	884 (67%)
Naji P. (2014), USA [27]	Retrospective, single-center	Treadmill exercise echocardiography	Standard Bruce protocol	Asymptomatic or mildly symptomatic ≥ grade III myxomatous MR	576 (70%)
Naji P. (2015), USA [28]	Retrospective, single-center	Treadmill exercise echocardiography	Standard Bruce protocol	Asymptomatic or mildly symptomatic ≥ grade III myxomatous MR	609 (67%)
Magne J. (2015), Belgium/France/Canada [29]	Prospective, multicenter	Semi-supine bicycle ergometer	25 W start, +25 W every 2 min	Asymptomatic or mildly symptomatic primary MR, LVEF > 60%, no LV dilation	102 (68%)
Lancellotti P. (2015), Belgium [30]	Prospective, single-center	Semi-supine bicycle ergometer	25 W start, +25 W every 2 min	Secondary MR with LV systolic dysfunction, sinus rhythm	159 (66%)
Mentias A. (2016), USA [31]	Retrospective, single-center	Treadmill exercise echocardiography	Bruce or modified Bruce; symptom-limited	Severe primary MR (≥3+), preserved LVEF ≥ 60%	737 (68%)
Bandera F. (2017), Italy [32]	Prospective, single-center	Semi-supine bicycle with CPET	Individualized ramp (≤12 W/min increase)	HFrEF (LVEF ≤ 40%), dilated LV, mainly ischemic	102 (71%)
Park S.J. (2017), Korea [33]	Prospective, single-center	Treadmill exercise echocardiography	Bruce protocol	Moderate-to-severe primary MR, LVEF > 60%, LVESD < 40 mm	114 (57%)
Vitel E. (2018), France [34]	Prospective, single-center	Semi-supine bicycle ergometer	30 W start, +30 W every 2 min	Isolated severe primary MR, preserved LVEF	142 (68%)
Suzuki T. (2019), Japan [35]	Prospective, single-center	Semi-supine bicycle ergometer	10 W start, +10 W every 3 min	Secondary MR, LVEF < 50%	118 (76%)
Izumo M. (2021), Japan [36]	Retrospective, single-center	Semi-supine bicycle ergometer	10 W start, +10 W every 3 min	Exercise-induced secondary MR (EROA ≥ 0.13 cm^2^)	46 (66%)
Sonaglioni A. (2022), Italy [37]	Retrospective, single-center	Semi-supine bicycle ergometer	25 W start, +25 W every 2 min	Symptomatic moderate primary MR (MVP)	424 (48%)
Peteiro J. (2022), Spain [38]	Prospective, single-center	Treadmill exercise echocardiography	Bruce (75%) or modified protocols (25%); symptom-limited	LVEF ≥ 50%, less than moderate MR	772 (48%)
Fino C. (2025), Italy [39]	Prospective, single-center	Semi-supine bicycle ergometer	Incremental until symptom-limited peak	Significant ischemic MR undergoing surgery	50 (66%)

Data are presented as reported in the original studies. Study design, stress modality, workload protocol, and population characteristics are summarized for each included study. CPET, cardiopulmonary exercise testing; EROA, effective regurgitant orifice area; LV, left ventricle; LVEF, left ventricular ejection fraction; LVESD, left ventricular end-systolic diameter; MR, mitral regurgitation; MVP, mitral valve prolapse; NYHA, New York Heart Association.

**Table 2:** Baseline clinical characteristics, cardiovascular risk factors, and medical therapy of the pooled population.

Parameter	Weighted Median	Weighted IQR (Q1–Q3)	Studies Included	Size (n)
Age (years)	58.5	57.0–64.0	19	5555
Male sex (%)	67.0	60.0–70.0	19	5555
BSA (m^2^)	1.84	1.77–1.85	8	2371
BMI (kg/m^2^)	25.7	24.0–27.5	6	3061
Hypertension (%)	47.0	35.0–67.0	17	5366
Diabetes mellitus (%)	4.0	4.0–19.2	17	5366
Smoking (%)	31.4	21.0–49.0	12	3149
Dyslipidemia (%)	42.2	30.0–58.0	13	3199
Obesity (%)	46.0	34.0–58.0	2	228
Coronary artery disease (%)	11.1	5.0–31.0	12	4474
Chronic kidney disease (%)	4.0	0.0–7.0	3	198
Atrial fibrillation (%)	11.1	5.0–27.0	14	4341
Prior stroke (%)	2.0	2.0–5.0	5	2856
Antiplatelets (%)	34.9	25.0–59.0	4	2493
Anticoagulants (%)	6.8	6.8–6.8	1	424
ACEi/ARBs (%)	38.4	30.0–50.0	17	5338
Beta-blockers (%)	30.7	25.0–41.0	16	5015
Diuretics (%)	12.9	8.0–28.0	11	1966
Mineralocorticoid receptor antagonists (%)	24.2	15.0–35.0	3	359
Calcium channel blockers (%)	8.3	5.0–12.0	3	1209
Nitrates (%)	8.5	5.0–30.0	6	1504
Statins (%)	26.1	20.0–56.0	2	526
Digoxin (%)	0.5	0.5–13.0	3	1166
STS score (%)	1.5	1.5–1.5	3	833
BNP (pg/mL)	48.0	40.0–56.8	2	228
NT-proBNP (pg/mL)	221.8	221.8–2226	3	262

Data are presented as weighted medians with corresponding interquartile ranges (IQR; Q1–Q3), with weighting based on the sample size of each study. The column “Studies included” refers to the number of studies contributing data for each variable, while “Size (n)” indicates the overall number of patients considered for that specific parameter. ACEi, angiotensin-converting enzyme inhibitors; ARBs, angiotensin II receptor blockers; BMI, body mass index; BNP, brain natriuretic peptide; BSA, body surface area; IQR, interquartile range; NT-proBNP, N-terminal pro–brain natriuretic peptide; Q1, first quartile; Q3, third quartile; STS, Society of Thoracic Surgeons.

**Table 3:** Resting and exercise hemodynamic and echocardiographic parameters.

Parameter	Rest	Peak Exercise	Δ (Exercise–Rest)	Studies (n)
Heart rate (bpm)	74.3 (71.2–76.7)	136.7 (121.2–142.0)	56.9 (49.8–61.1)	8 (1741)
SBP (mmHg)	129.3 (128.1–134.6)	174.0 (157.2–174.3)	41.7 (25.5–43.5)	8 (1741)
DBP (mmHg)	77.7 (75.4–81.0)	80.7 (79.0–83.8)	6.6 (5.3–8.9)	4 (437)
RWT	0.3 (0.3–0.4)	—	—	2 (526)
LVMi (g/m^2^)	97.3 (97.3–104.0)	—	—	3 (640)
LVEDD (mm)	49.7 (47.2–50.3)	—	—	12 (3548)
LVESD (mm)	30.0 (29.2–30.9)	—	—	12 (3548)
LVEDV (mL)	121.7 (73.0–148.2)	141.6 (110.9–171.3)	6.1 (5.3–7.1)	9 (1181)
LVESV (mL)	92.6 (45.6–111.9)	79.9 (34.9–95.2)	—	8 (757)
LVEF (%)	58.0 (57.0–60.7)	60.3 (39.0–63.4)	2.6 (2.5–4.7)	18 (5440)
SV (mL)	53.7 (46.4–54.4)	55.4 (51.9–59.0)	4.0 (4.0–5.7)	3 (198)
CO (L/min)	3.5 (3.2–3.8)	5.7 (4.7–6.2)	2.5 (0.4–2.6)	3 (198)
LV-GLS (%)	20.8 (20.2–21.4)	22.0 (22.0–22.2)	−0.3 (−2.4–1.9)	3 (992)
EROA (cm^2^)	0.44 (0.20–0.47)	0.52 (0.26–0.55)	0.08 (0.07–0.09)	15 (3505)
Regurgitant volume (mL)	67.9 (66.0–68.0)	75.0 (36.5–75.0)	7.0 (5.6–9.9)	14 (3228)
Moderate–severe MR (%)	63.0 (29.3–66.4)	68.6 (52.0–81.0)	—	14 (4754)
E/A	0.9 (0.9–0.9)	—	—	2 (526)
E/e’	11.4 (9.6–14.1)	12.2 (11.7–14.6)	3.9 (1.1–4.2)	9 (1951)
LAVi (mL/m^2^)	42.2 (24.4–49.2)	42.5 (38.4–44.3)	1.8 (1.8–1.9)	9 (1224)
TAPSE (mm)	19.7 (17.6–21.8)	26.3 (19.8–27.5)	4.7 (2.6–5.5)	5 (764)
sPAP (mmHg)	31.0 (30.6–34.1)	46.4 (46.0–50.3)	16.3 (15.0–17.8)	17 (5161)
TAPSE/sPAP	0.60 (0.50–0.60)	0.40 (0.40–0.40)	—	3 (238)
METs	—	9.6 (8.3–9.8)	—	8 (4086)

Data are presented as weighted medians with corresponding interquartile ranges (IQR; Q1–Q3), with weighting based on the sample size of each study. The column “Studies (n)” refers to the number of studies contributing data for each variable, while “Size (n)” indicates the overall number of patients included for that specific parameter. Δ indicates the difference between peak exercise and rest. CO, cardiac output; DBP, diastolic blood pressure; E/A, ratio of early to late diastolic transmitral flow velocity; E/e’, ratio of early transmitral flow velocity to early diastolic mitral annular velocity; EROA, effective regurgitant orifice area; LAVi, left atrial volume index; LVEDD, left ventricular end-diastolic diameter; LVEDV, left ventricular end-diastolic volume; LVEF, left ventricular ejection fraction; LVESD, left ventricular end-systolic diameter; LVESV, left ventricular end-systolic volume; LV-GLS, left ventricular global longitudinal strain; LVMi, left ventricular mass index; METs, metabolic equivalents; MR, mitral regurgitation; RWT, relative wall thickness; SBP, systolic blood pressure; sPAP, systolic pulmonary artery pressure; SV, stroke volume; TAPSE, tricuspid annular plane systolic excursion.

**Table 4:** Clinical Outcomes, Follow-Up Duration, and Main Prognostic Predictors Across Included Studies [21,22,23,24,25,26,27,28,29,30,31,32,33,34,35,36,37,38,39].

Study Name	Follow-Up (Months)	Events (Rate %)	Endpoint	Main Predictors
Lancellotti P. [21]	19 (8)	9 (11%)	Cardiac death	ΔEROA ≥ 0.13 cm^2^; resting EROA ≥ 0.20 cm^2^; shorter mitral deceleration time
Lee R. [22]	36 (12)	8 (11%)	Composite of cardiac death, heart failure, and new-onset AF	Absence of LV contractile reserve
Peteiro J. [23]	20.4 (18)	43 (13%)	Hard cardiac events (cardiac death and nonfatal MI)	Resting MR; peak HR × SBP; extent of coronary disease
Magne J. [24]	23 (19)	46 (41%)	Composite of cardiovascular death, heart failure hospitalization, or mitral valve surgery	Exercise BNP
Magne J. [25]	24 (21)	47 (41%)	Composite of cardiovascular death, mitral valve surgery, or heart failure/pulmonary edema	Absence of LV contractile reserve assessed by GLS
Naji P. [26]	76.8 (48)	87 (10%)	Composite of death, myocardial infarction, stroke, or progression to heart failure	Heart rate recovery; % predicted METs; resting RVSP; atrial fibrillation; resting LVEF
Naji P. [27]	79 (48)	53 (9%)	Composite of death, myocardial infarction, stroke, or heart failure progression	Age; % predicted METs; resting LVEF
Naji P. [28]	64	120 (20%)	Composite of death or progression to heart failure	Atrial fibrillation; resting LVEF; RVSP; holosystolic MR; % predicted METs
Magne J. [29]	50 (23)	28 (27%)	Composite cardiovascular events (death, hospitalization, stroke, AF)	Exercise pulmonary hypertension
Lancellotti P. [30]	35 (11)	55 (35%)	Combined cardiac events (HF hospitalization, death, device implantation, transplant)	Exercise pulmonary hypertension
Mentias A. [31]	99.6 (36)	64 (9%)	All-cause mortality	STS score; LV-GLS; RVSP; % predicted METs; mitral valve surgery
Bandera F. [32]	12.8 (6.8)	8 (10%)	Composite of death or heart failure hospitalization	Resting severe MR; exercise-induced severe MR
Park S.J. [33]	42 (18)	39 (34%)	Composite of mitral valve surgery or new LV systolic dysfunction	NT-proBNP; contractile reserve; atrial fibrillation; resting EROA; RV size
Vitel E. [34]	30	48 (34%)	Major adverse cardiovascular events (AF, stroke, hospitalization, or death)	Exercise TAPSE; FAC; male sex; RV size; RV strain
Suzuki T. [35]	41.7	49 (42%)	Major adverse cardiac events (cardiac death or HF hospitalization)	Exercise EROA; age
Izumo M. [36]	13	11 (24%)	Composite of death or heart failure hospitalization	Transcatheter mitral repair; exercise LVEF
Sonaglioni A. [37]	38.4 (20.4)	75 (18%)	Composite of cardiovascular hospitalization, mitral valve surgery, or cardiac death	Age; diabetes mellitus; peak exercise E/e’; peak exercise EROA; MHI; beta-blocker therapy
Peteiro J. [38]	20.2	132 (17%)	Composite of death, myocardial infarction, hospitalization, or revascularization	Peak exercise LVEF; exercise E/e’
Fino C. [39]	120	21 (42%)	Preoperative adverse events based on exercise RV–pulmonary coupling	Exercise TAPSE/sPAP < 0.34

Follow-up duration, event rates, endpoint definitions, and main prognostic predictors identified in each study are reported. AF, atrial fibrillation; BNP, brain natriuretic peptide; EROA, effective regurgitant orifice area; FAC, fractional area change; GLS, global longitudinal strain; HF, heart failure; HR, heart rate; LVEF, left ventricular ejection fraction; METs, metabolic equivalents; MI, myocardial infarction; MHI, modified Haller index; MR, mitral regurgitation; NT-proBNP, N-terminal pro–brain natriuretic peptide; RV, right ventricle; RVSP, right ventricular systolic pressure; sPAP, systolic pulmonary artery pressure; STS, Society of Thoracic Surgeons; TAPSE, tricuspid annular plane systolic excursion.

**Table 5:** Methodological Quality Assessment of Included Studies [21,22,23,24,25,26,27,28,29,30,31,32,33,34,35,36,37,38,39] According to the NIH Tool.

Study Name	Q1	Q2	Q3	Q4	Q5	Q6	Q7	Q8	Q9	Q10	Q11	Q12	Q13	Q14	Overall
Lancellotti P. [21]	Y	Y	NR	Y	NR	Y	Y	Y	Y	N	Y	NR	Y	Y	Good
Lee R. [22]	Y	Y	NR	Y	NR	Y	Y	Y	Y	N	Y	NR	Y	Y	Good
Peteiro J. [23]	Y	Y	NR	Y	NR	Y	Y	Y	Y	N	Y	NR	Y	Y	Good
Magne J. [24]	Y	Y	NR	Y	NR	Y	Y	Y	Y	N	Y	NR	Y	Y	Good
Magne J. [25]	Y	Y	NR	Y	NR	Y	Y	Y	Y	N	Y	NR	Y	Y	Good
Naji P. [26]	Y	Y	Y	Y	Y	Y	Y	Y	Y	N	Y	NR	Y	Y	Good
Naji P. [27]	Y	Y	Y	Y	Y	Y	Y	Y	Y	N	Y	NR	Y	Y	Good
Naji P. [28]	Y	Y	NR	Y	NR	Y	Y	Y	Y	N	Y	NR	Y	Y	Good
Magne J. [29]	Y	Y	NR	Y	NR	Y	Y	Y	Y	N	Y	NR	Y	Y	Good
Lancellotti P. [30]	Y	Y	Y	Y	Y	Y	Y	Y	Y	N	Y	NR	Y	Y	Good
Mentias A. [31]	Y	Y	Y	Y	Y	Y	Y	Y	Y	N	Y	NR	Y	Y	Good
Bandera F. [32]	Y	Y	NR	Y	NR	Y	Y	Y	Y	N	Y	NR	Y	Y	Good
Park S.J. [33]	Y	Y	NR	Y	NR	Y	Y	Y	Y	N	Y	NR	Y	Y	Good
Vitel E. [34]	Y	Y	NR	Y	NR	Y	Y	Y	Y	N	Y	NR	Y	Y	Good
Suzuki T. [35]	Y	Y	NR	Y	NR	Y	Y	Y	Y	N	Y	NR	Y	Y	Good
Izumo M. [36]	Y	Y	NR	Y	NR	Y	Y	Y	Y	N	Y	NR	NR	Y	Good
Sonaglioni A. [37]	Y	Y	NR	Y	NR	Y	Y	Y	Y	N	Y	NR	NR	Y	Good
Peteiro J. [38]	Y	Y	Y	Y	NR	Y	Y	Y	Y	N	Y	NR	Y	Y	Good
Fino C. [39]	Y	Y	NR	Y	NR	Y	Y	Y	Y	N	Y	NR	NR	Y	Good

Q1–Q14 represent individual quality criteria domains. N, no; NIH, National Institutes of Health; NR, not reported; Q, question; Y, yes.

## Data Availability

Data extracted from included studies will be publicly available on Zenodo (https://zenodo.org) (accessed on 9 April 2026).

## Supplementary Materials

The following supporting information can be downloaded at: https://www.mdpi.com/article/10.3390/jcm15093253/s1, File S1: PRISMA 2020 checklist; File S2: The full INPLASY record [1,2,3,4,5,6,7,8,9,10,11,12,13,14,15,16,17,18] (https://inplasy.com/inplasy-2026-4-0023/, accessed on 7 April 2026).

## References

[1] Figlioli G., Sticchi A., Christodoulou M.N., Hadjidemetriou A., Amorim Moreira Alves G., De Carlo M., Praz F., Caterina R., Nikolopoulos G.K., Bonovas S. (2025). Global Prevalence of Mitral Regurgitation: A Systematic Review and Meta-Analysis of Population-Based Studies. J. Clin. Med..

[2] Neema P.K., Panidapu N. (2025). The Mechanisms and Pathophysiology of Mitral Regurgitation: A Narrative Review. Ann. Card. Anaesth..

[3] Saeed M., Sabanci R., Ghnaima H., Watat K., Shaban D., Nader G., Banga S., Wilcox M. (2024). Navigating Asymptomatic Mitral Regurgitation: Diagnostic Dilemmas and Treatment Strategies. Cureus.

[4] Delgado V., Ajmone Marsan N., Bonow R.O., Hahn R.T., Norris R.A., Zühlke L., Borger M.A. (2023). Degenerative mitral regurgitation. Nat. Rev. Dis. Primers.

[5] Pastore M.C., Mandoli G.E., Dokollari A., Bisleri G., D’Ascenzi F., Santoro C., Miglioranza M.H., Focardi M., Cavigli L., Patti G. (2022). Speckle tracking echocardiography in primary mitral regurgitation: Should we reconsider the time for intervention?. Heart Fail. Rev..

[6] Messika-Zeitoun D., Chu M.W.A., Bouchard D., Le Tourneau T., Ternacle J., Demers P., Guo L., Fu A.Y.N., Dib J.C., Lam C. (2025). Clinical Presentation and Outcomes After Surgery for Mitral Regurgitation: Real-World Insights From the MITRACURE International Registry. Circulation.

[7] Marchetti D., Di Lenarda F., Novembre M.L., Paolisso P., Schillaci M., Melotti E., Doldi M., Terzi R., Gallazzi M., Conte E. (2023). Contemporary Echocardiographic Evaluation of Mitral Regurgitation and Guidance for Percutaneous Mitral Valve Repair. J. Clin. Med..

[8] Rolando M., Elmasry N., Gobbi F., Moreo A., Ajmone Marsan N., Carluccio E., Fortuni F. (2025). Echocardiographic Assessment of Patients Undergoing Mitral Valve Repair. J. Cardiovasc. Dev. Dis..

[9] Leo L.A., Viani G., Schlossbauer S., Bertola S., Valotta A., Crosio S., Pasini M., Caretta A. (2025). Mitral Regurgitation Evaluation in Modern Echocardiography: Bridging Standard Techniques and Advanced Tools for Enhanced Assessment. Echocardiography.

[10] Duncan C.F., Bowcock E., Pathan F., Orde S.R. (2023). Mitral regurgitation in the critically ill: The devil is in the detail. Ann. Intensive Care.

[11] Vilela E.M., Sampaio F., Ribeiro J., Fontes-Carvalho R. (2026). Exercise Stress Echocardiography: A Dynamic Assessment for an Evolving Landscape. Rev. Cardiovasc. Med..

[12] Correra A., Mauriello A., Del Giudice C., Fonderico C., Di Peppo M., Russo V., D’Andrea A., Esposito G., Brunetti N.D. (2026). The Incremental Role of Stress Echocardiography in Valvular Heart Disease: A Narrative Review. Diagnostics.

[13] Ueyama H., Kuno T., Takagi H., Krishnamoorthy P., Prandi F.R., Palazzuoli A., Sharma S.K., Kini A., Lerakis S. (2023). Prognostic value of left ventricular global longitudinal strain in mitral regurgitation: A systematic review. Heart Fail. Rev..

[14] Moura-Ferreira S., Pugliese N.R., Milani M., Taddei S., Jacobs A., De Biase N., Dhont S., Falter M., Bekhuis Y., L’Hoyes W. (2025). Prognostic Value of Exercise Right Ventricular-Pulmonary Arterial Coupling in Primary Mitral Regurgitation. Circulation.

[15] Citro R., Bursi F., Bellino M., Picano E. (2022). The Role of Stress Echocardiography in Valvular Heart Disease. Curr. Cardiol. Rep..

[16] Altes A., Vermes E., Levy F., Vancraeynest D., Pasquet A., Vincentelli A., Gerber B.L., Tribouilloy C., Maréchaux S. (2023). Quantification of primary mitral regurgitation by echocardiography: A practical appraisal. Front. Cardiovasc. Med..

[17] Wu Q., Wang L., Li Y., Liang Z., Li Q., Liu X., Yin Y., Liu Y., Hu Z., Gao H. (2025). Prognostic impact of mitral regurgitation in elderly patients with atrial fibrillation: Results from the CABANA trial. Open Heart.

[18] Gallo G., Forte M., Stanzione R., Cotugno M., Bianchi F., Marchitti S., Berni A., Volpe M., Rubattu S. (2020). Functional Role of Natriuretic Peptides in Risk Assessment and Prognosis of Patients with Mitral Regurgitation. J. Clin. Med..

[19] Page M.J., McKenzie J.E., Bossuyt P.M., Boutron I., Hoffmann T.C., Mulrow C.D., Shamseer L., Tetzlaff J.M., Akl E.A., Brennan S.E. (2021). The PRISMA 2020 statement: An updated guideline for reporting systematic reviews. BMJ.

[20] Ma L.L., Wang Y.Y., Yang Z.H., Huang D., Weng H., Zeng X.T. (2020). Methodological quality (risk of bias) assessment tools for primary and secondary medical studies: What are they and which is better?. Mil. Med. Res..

[21] Lancellotti P., Troisfontaines P., Toussaint A.C., Pierard L.A. (2003). Prognostic importance of exercise-induced changes in mitral regurgitation in patients with chronic ischemic left ventricular dysfunction. Circulation.

[22] Lee R., Haluska B., Leung D.Y., Case C., Mundy J., Marwick T.H. (2005). Functional and prognostic implications of left ventricular contractile reserve in patients with asymptomatic severe mitral regurgitation. Heart.

[23] Peteiro J., Bendayan I., Mariñas J., Campos R., Bouzas B., Castro-Beiras A. (2008). Prognostic value of mitral regurgitation assessment during exercise echocardiography in patients with left ventricular dysfunction: A follow-up study of 1.7 +/− 1.5 years. Eur. J. Echocardiogr..

[24] Magne J., Mahjoub H., Pibarot P., Pirlet C., Pierard L.A., Lancellotti P. (2012). Prognostic importance of exercise brain natriuretic peptide in asymptomatic degenerative mitral regurgitation. Eur. J. Heart Fail..

[25] Magne J., Mahjoub H., Dulgheru R., Pibarot P., Pierard L.A., Lancellotti P. (2014). Left ventricular contractile reserve in asymptomatic primary mitral regurgitation. Eur. Heart J..

[26] Naji P., Griffin B.P., Asfahan F., Barr T., Rodriguez L.L., Grimm R., Agarwal S., Stewart W.J., Mihaljevic T., Gillinov A.M. (2014). Predictors of long-term outcomes in patients with significant myxomatous mitral regurgitation undergoing exercise echocardiography. Circulation.

[27] Naji P., Griffin B.P., Barr T., Asfahan F., Gillinov A.M., Grimm R.A., Rodriguez L.L., Mihaljevic T., Stewart W.J., Desai M.Y. (2014). Importance of exercise capacity in predicting outcomes and determining optimal timing of surgery in significant primary mitral regurgitation. J. Am. Heart Assoc..

[28] Naji P., Asfahan F., Barr T., Rodriguez L.L., Grimm R.A., Agarwal S., Thomas J.D., Gillinov A.M., Mihaljevic T., Griffin B.P. (2015). Impact of duration of mitral regurgitation on outcomes in asymptomatic patients with myxomatous mitral valve undergoing exercise stress echocardiography. J. Am. Heart Assoc..

[29] Magne J., Donal E., Mahjoub H., Miltner B., Dulgheru R., Thebault C., Pierard L.A., Pibarot P., Lancellotti P. (2015). Impact of exercise pulmonary hypertension on postoperative outcome in primary mitral regurgitation. Heart.

[30] Lancellotti P., Magne J., Dulgheru R., Ancion A., Martinez C., Piérard L.A. (2015). Clinical significance of exercise pulmonary hypertension in secondary mitral regurgitation. Am. J. Cardiol..

[31] Mentias A., Naji P., Gillinov A.M., Rodriguez L.L., Reed G., Mihaljevic T., Suri R.M., Sabik J.F., Svensson L.G., Grimm R.A. (2016). Strain Echocardiography and Functional Capacity in Asymptomatic Primary Mitral Regurgitation with Preserved Ejection Fraction. J. Am. Coll. Cardiol..

[32] Bandera F., Generati G., Pellegrino M., Garatti A., Labate V., Alfonzetti E., Gaeta M., Castelvecchio S., Menicanti L., Guazzi M. (2017). Mitral regurgitation in heart failure: Insights from CPET combined with exercise echocardiography. Eur. Heart J. Cardiovasc. Imaging.

[33] Park S.J., Cho E.J., Ahn J., Carriere K., Kim E.K., Lee G.Y., Chang S.A., Choi J.O., Lee S.C., Park S.W. (2017). Additive prognostic values of NT-proBNP and exercise stress echocardiography in asymptomatic patients with degenerative mitral regurgitation and preserved left ventricular ejection fraction. Int. J. Cardiol..

[34] Vitel E., Galli E., Leclercq C., Fournet M., Bosseau C., Corbineau H., Bouzille G., Donal E. (2018). Right ventricular exercise contractile reserve and outcomes after early surgery for primary mitral regurgitation. Heart.

[35] Suzuki T., Izumo M., Suzuki K., Koto D., Tsukahara M., Teramoto K., Sato Y., Watanabe M., Mizukoshi K., Kamijima R. (2019). Prognostic value of exercise stress echocardiography in patients with secondary mitral regurgitation: A long-term follow-up study. J. Echocardiogr..

[36] Izumo M., Kuwata S., Ishibashi Y., Suzuki T., Ohara H., Watanabe M., Sato Y., Nishikawa H., Okuyama K., Kamijima R. (2021). Prognostic impact of transcatheter mitral valve repair in patients with exercise-induced secondary mitral regurgitation. Eur. Heart J. Cardiovasc. Imaging.

[37] Sonaglioni A., Nicolosi G.L., Rigamonti E., Lombardo M. (2022). Impact of Chest Wall Conformation on the Outcome of Primary Mitral Regurgitation due to Mitral Valve Prolapse. J. Cardiovasc. Echogr..

[38] Peteiro J., Bouzas-Mosquera A., Barbeito-Caamaño C., Martin-Alvarez E., Souto-Cainzos B., Vazquez-Rodriguez J.M. (2022). Additive prognostic and diagnostic value of diastolic exercise parameters in patients referred for exercise echocardiography. Eur. Heart J. Cardiovasc. Imaging.

[39] Fino C., Bellavia D., D’Alonzo M., Merlo M., Bruno V.D., Magne J., Caputo M., Terzi A., Senni M., Bichi S. (2025). Exercise Right Ventricular-Pulmonary Arterial Coupling and Functional Outcome in Patients Undergoing Surgery for Secondary Ischemic Mitral Regurgitation. J. Am. Heart Assoc..

[40] Sugimoto T., Bandera F., Generati G., Alfonzetti E., Barletta M., Losito M., Labate V., Rovida M., Caracciolo M., Pappone C. (2020). Left Atrial Dynamics During Exercise in Mitral Regurgitation of Primary and Secondary Origin: Pathophysiological Insights by Exercise Echocardiography Combined with Gas Exchange Analysis. JACC Cardiovasc. Imaging.

[41] Magne J., Lancellotti P., Piérard L.A. (2010). Exercise-induced changes in degenerative mitral regurgitation. J. Am. Coll. Cardiol..

[42] Gaasch W.H., Meyer T.E. (2008). Left ventricular response to mitral regurgitation: Implications for management. Circulation.

[43] McCutcheon K., Manga P. (2018). Left ventricular remodelling in chronic primary mitral regurgitation: Implications for medical therapy. Cardiovasc. J. Afr..

[44] Lancellotti P., Magne J. (2012). Stress testing for the evaluation of patients with mitral regurgitation. Curr. Opin. Cardiol..

[45] Antoine C., Benfari G., Michelena H.I., Maalouf J.F., Nkomo V.T., Thapa P., Enriquez-Sarano M. (2018). Clinical Outcome of Degenerative Mitral Regurgitation: Critical Importance of Echocardiographic Quantitative Assessment in Routine Practice. Circulation.

[46] Karam N., Orban M., Kalbacher D., Butter C., Praz F., Lubos E., Bannehr M., Kassar M., Petrescu A., Iliadis C. (2021). Impact of effective regurgitant orifice area on outcome of secondary mitral regurgitation transcatheter repair. Clin. Res. Cardiol..

[47] Spieker M., Sidabras J., Lagarden H., Christian L., Günther N., Angendohr S., Bejinariu A., Schulze P.C., Pfister R., Öztürk C. (2025). Exercise-induced dynamic mitral regurgitation is associated with outcomes in patients with ischaemic cardiomyopathy. ESC Heart Fail..

[48] Moss R.R., Bar S.L., Chandavimol M., Munt B., Thompson C.R., Abel J.G., Humphries K., Ignaszewski A.P. (2014). Contractile reserve induced with dobutamine echocardiography predicts outcome in patients with left ventricular dysfunction and mitral regurgitation. J. Heart Valve Dis..

[49] Lancellotti P., Cosyns B., Zacharakis D., Attena E., Van Camp G., Gach O., Radermecker M., Piérard L.A. (2008). Importance of left ventricular longitudinal function and functional reserve in patients with degenerative mitral regurgitation: Assessment by two-dimensional speckle tracking. J. Am. Soc. Echocardiogr..

[50] De Luca A., Stolfo D., Caiffa T., Korcova R., Barbati G., Vitrella G., Rakar S., Perkan A., Secoli G., Pinamonti B. (2019). Prognostic Value of Global Longitudinal Strain-Based Left Ventricular Contractile Reserve in Candidates for Percutaneous Correction of Functional Mitral Regurgitation: Implications for Patient Selection. J. Am. Soc. Echocardiogr..

[51] Xie P., Zhou H., Huang Y., Huang Z., Liu M., Hui Z., Guo Y., Li S., Fan R., Xiong Z. (2026). Left ventricular diastolic function modifies clinical outcomes of mild mitral regurgitation. Int. J. Cardiol..

[52] Lababidi H., El-Tallawi K.C., Kitkungvan D., Angulo C.L., Shah D.J., Zoghbi W.A., Nagueh S.F. (2025). The prognostic importance of left ventricular diastolic function in primary mitral regurgitation and its relation to structural changes by CMR. Sci. Rep..

[53] Nagueh S.F., Smiseth O.A., Appleton C.P., Byrd B.F., Dokainish H., Edvardsen T., Flachskampf F.A., Gillebert T.C., Klein A.L., Lancellotti P. (2016). Recommendations for the Evaluation of Left Ventricular Diastolic Function by Echocardiography: An Update from the American Society of Echocardiography and the European Association of Cardiovascular Imaging. J. Am. Soc. Echocardiogr..

[54] Peikert A., Fontana M., Solomon S.D., Thum T. (2026). Left ventricular hypertrophy and myocardial fibrosis in heart failure with preserved ejection fraction: Mechanisms and treatment. Eur. Heart J..

[55] Velidakis N., Khattab E., Gkougkoudi E., Kadoglou N.P.E. (2023). Pulmonary Hypertension in Left Ventricular Valvular Diseases: A Comprehensive Review on Pathophysiology and Prognostic Value. Life.

[56] Ho J.E., Zern E.K., Lau E.S., Wooster L., Bailey C.S., Cunningham T., Eisman A.S., Hardin K.M., Farrell R., Sbarbaro J.A. (2020). Exercise Pulmonary Hypertension Predicts Clinical Outcomes in Patients with Dyspnea on Effort. J. Am. Coll. Cardiol..

[57] Kadoglou N.P.E., Papadopoulos C.H., Krommydas A. (2022). The prognostic value of exercise-induced pulmonary hypertension in asymptomatic patients with primary mitral regurgitation. J. Cardiol..

[58] Karam N., Stolz L., Orban M., Deseive S., Praz F., Kalbacher D., Westermann D., Braun D., Näbauer M., Neuss M. (2021). Impact of Right Ventricular Dysfunction on Outcomes After Transcatheter Edge-to-Edge Repair for Secondary Mitral Regurgitation. JACC Cardiovasc. Imaging.

[59] Chehab O., Long E., Androshchuk V., Gill H., Avlonitis V., Bosco P., Lucchese G., Patterson T., Redwood S., Rajani R. (2024). Right ventricular to pulmonary arterial coupling as a predictor of survival in patients undergoing mitral valve surgery for mitral regurgitation. Eur. J. Cardiothorac. Surg..

[60] Praz F., Borger M.A., Lanz J., Marin-Cuartas M., Abreu A., Adamo M., Ajmone Marsan N., Barili F., Bonaros N., Cosyns B. (2025). 2025 ESC/EACTS Guidelines for the management of valvular heart disease. Eur. Heart J..

[61] Sonaglioni A., Baravelli M., Vincenti A., Trevisan R., Zompatori M., Nicolosi G.L., Lombardo M., Anzà C. (2018). A New modified anthropometric haller index obtained without radiological exposure. Int. J. Cardiovasc. Imaging.

[62] Sonaglioni A., Nicolosi G.L., Muti-Schünemann G.E.U., Rispoli G.A., Lombardo M., Muti P. (2025). Does Preliminary Chest Shape Assessment Improve the Prognostic Risk Stratification of Individuals with Mitral Annular Disjunction? A Case Report and Narrative Review. J. Clin. Med..

[63] Sonaglioni A., Nicolosi G.L., Lombardo M., Gensini G.F., Ambrosio G. (2021). Influence of chest conformation on myocardial strain parameters in healthy subjects with mitral valve prolapse. Int. J. Cardiovasc. Imaging.

[64] Sonaglioni A., Grasso E., Nicolosi G.L., Lombardo M. (2024). Modified Haller Index is inversely associated with asymptomatic status in atrial fibrillation patients undergoing electrical cardioversion: A preliminary observation. Minerva Cardiol. Angiol..

[65] Lee C., Dow S., Shah K., Henkin S., Taub C. (2023). Complications of exercise and pharmacologic stress echocardiography. Front. Cardiovasc. Med..

[66] Woodward W., Dockerill C., McCourt A., Upton R., O’Driscoll J., Balkhausen K., Chandrasekaran B., Firoozan S., Kardos A., Wong K. (2022). Real-world performance and accuracy of stress echocardiography: The EVAREST observational multi-centre study. Eur. Heart J. Cardiovasc. Imaging.

[67] Sonaglioni A., Polymeropoulos A., Baravelli M., Nicolosi G.L., Lombardo M., Biondi-Zoccai G. (2025). Diagnostic Accuracy of Exercise Stress Testing, Stress Echocardiography, Myocardial Scintigraphy, and Cardiac Magnetic Resonance for Obstructive Coronary Artery Disease: Systematic Reviews and Meta-Analyses of 104 Studies Published from 1990 to 2025. J. Clin. Med..

[68] Hagendorff A., Knebel F., Helfen A., Stöbe S., Haghi D., Ruf T., Lavall D., Knierim J., Altiok E., Brandt R. (2021). Echocardiographic assessment of mitral regurgitation: Discussion of practical and methodologic aspects of severity quantification to improve diagnostic conclusiveness. Clin. Res. Cardiol..

[69] Nicolosi G.L. (2020). The strain and strain rate imaging paradox in echocardiography: Overabundant literature in the last two decades but still uncertain clinical utility in an individual case. Arch. Med. Sci. Atheroscler. Dis..

[70] Santoro C., Donal E., Magne J., Sade L.E., Penicka M., Katbeh A., Cosyns B., Cameli M., Hanzevacki J.S., Luksic V.R. (2023). Inter-center reproducibility of standard and advanced echocardiographic parameters in the EACVI-AFib echo registry. Echocardiography.

[71] Kadoglou N.P.E., Papadopoulos C.H., Papadopoulos K.G., Karagiannis S., Karabinos I., Loizos S., Theodosis-Georgilas A., Aggeli K., Keramida K., Klettas D. (2022). Updated knowledge and practical implementations of stress echocardiography in ischemic and non-ischemic cardiac diseases: An expert consensus of the Working Group of Echocardiography of the Hellenic Society of Cardiology. Hell. J. Cardiol..

[72] Di Fiore V., Del Punta L., De Biase N., Masi S., Taddei S., Rosada J., Emdin M., Passino C., Fabiani I., Pugliese N.R. (2025). Advancing Cardiovascular Risk Stratification and Functional Assessment: A Narrative Review of CPET and ESE Applications. Healthcare.

[73] Tokarek T., Dziewierz A., Dudek D. (2021). MitraClip for mitral valve regurgitation and transcatheter aortic valve implantation for severe aortic valve stenosis: State-of-the-art. Postep. Kardiol. Interwencyjnej.

