# Supplementary material for: Unmasking Risk in Mitral Regurgitation: Prognostic Value of Exercise Stress Echocardiography—A Systematic Review
Source: J Clin Med. 2026 Apr 24;15(9):3253. doi: 10.3390/jcm15093253 (PMC13163529; doi:10.3390/jcm15093253)
Supplement: Supplementary file 1 [file jcm-15-03253-s001.zip › Supplementary Material S2.pdf]

International Platform of Registered Systematic Review and Meta-analysis Protocols

# INPLASY

## Unmasking Risk in Mitral Regurgitation: Prognostic Value of Exercise Stress Echocardiography—A Systematic Review

INPLASY202640023
doi: 10.37766/inplasy2026.4.0023
Received: 7 April 2026
Published: 7 April 2026

Sonaglioni, A; Baravelli, M; Gramaglia, GF; Nicolosi GL; Lombardo, M.

**Corresponding author:**
Andrea Sonaglioni

sonaglioniandrea@gmail.com

**Author Affiliation:**
IRCCS MultiMedica.

### ADMINISTRATIVE INFORMATION

**Support -** Ministero della Salute Ricerca Corrente.

**Review Stage at time of this submission -** Completed but not published.

**Conflicts of interest -** None declared.

**INPLASY registration number:** INPLASY202640023

**Amendments -** This protocol was registered with the International Platform of Registered Systematic Review and Meta-Analysis Protocols (INPLASY) on 7 April 2026 and was last updated on 7 April 2026.

### INTRODUCTION

**Review question / Objective** The present systematic review aims to provide an integrated overview of the available literature, focusing on exercise-derived echocardiographic and hemodynamic parameters associated with clinical outcomes. In addition, this work seeks to characterize the clinical profile of the studied populations and to describe the physiological response to exercise in mitral regurgitation (MR) , in order to better define the role of exercise stress echocardiography (ESE) in contemporary risk stratification and clinical decision-making.

**Rationale** Exercise stress echocardiography (ESE) offers a unique opportunity to evaluate MR within a dynamic framework. By integrating hemodynamic, functional, and structural information during exercise, ESE allows real-time assessment of regurgitation severity, ventricular performance, and cardiopulmonary interaction [11]. In particular, stress-induced changes in EROA and regurgitant volume, elevation of left ventricular filling pressures (e.g., E/e'), and increases in pulmonary artery pressure provide insight into the functional reserve of the cardiovascular system [12]. Additionally, indices of myocardial deformation such as global longitudinal strain (GLS) [13], as well as markers of right ventricular (RV)–pulmonary arterial (PA) coupling [e.g., tricuspid annular plane systolic excursion/systolic pulmonary artery pressure (TAPSE/sPAP)] [14], further contribute to a comprehensive evaluation of disease impact.
Over the past two decades, a substantial number of studies have explored the prognostic implications of ESE in MR across different clinical settings, including asymptomatic degenerative MR and functional MR associated with LV dysfunction [15]. These investigations have identified a wide array of potential predictors, ranging from exercise-induced worsening of regurgitation to impaired contractile reserve and abnormal hemodynamic responses. Notably, dynamic parameters such as exercise EROA, increase in regurgitant volume, elevated E/e', and exercise pulmonary hypertension have consistently

Sonaglioni et al. INPLASY protocol 202640023. doi:10.37766/inplasy2026.4.0023 Downloaded from https://inplasy.com/inplasy-2026-4-0023/

emerged as markers associated with adverse clinical outcomes [16] .
At the same time, clinical and functional variables—including age, atrial fibrillation, reduced exercise capacity, and biomarkers such as natriuretic peptides—have been shown to provide complementary prognostic information [17,18]. This highlights the multifactorial nature of risk in MR and the importance of integrating imaging findings with clinical context. However, the heterogeneity in study design, patient populations, stress protocols, and endpoint definitions has limited the generalizability of individual findings and hindered their translation into routine clinical practice. Moreover, the populations evaluated in previous studies are diverse, encompassing patients with preserved or reduced LVEF, primary and secondary MR, and varying degrees of symptom burden. This heterogeneity, while reflecting real-world clinical practice, further underscores the need for a structured synthesis of the available evidence.

References
11. Vilela, E.M.; Sampaio, F.; Ribeiro, J.; Fontes-Carvalho, R. Exercise Stress Echocardiography: A Dynamic Assessment for an Evolving Landscape. Rev. Cardiovasc. Med. 2026, 27, 47079. https://doi.org/10.31083/RCM47079.
12. Correra, A.; Mauriello, A.; Del Giudice, C.; Fonderico, C.; Di Peppo, M.; Russo, V.; D'Andrea, A.; Esposito, G.; Brunetti, N.D. The Incremental Role of Stress Echocardiography in Valvular Heart Disease: A Narrative Review. Diagnostics (Ba-sel) 2026, 16, 148. https://doi.org/10.3390/diagnostics16010148.
13. Ueyama, H.; Kuno, T.; Takagi, H.; Krishnamoorthy, P.; Prandi, F.R.; Palazzuoli, A.; Sharma, S.K.; Kini, A.; Lerakis, S. Prognostic value of left ventricular global longitudinal strain in mitral regurgitation: a systematic review. Heart Fail. Rev. 2023, 28, 465–483. https://doi.org/10.1007/s10741-022-10265-3.
14. Moura-Ferreira, S.; Pugliese, N.R.; Milani, M.; Taddei, S.; Jacobs, A.; De Biase, N.; Dhont, S.; Falter, M.; Bekhuis, Y.; L'Hoyes, W.; et al. Prognostic Value of Exercise Right Ventricular-Pulmonary Arterial Coupling in Primary Mitral Re-gurgitation. Circulation 2025, 152, 1594–1607. https://doi.org/10.1161/CIRCULATIONAHA.125.073778.
15. Citro, R.; Bursi, F.; Bellino, M.; Picano, E. The Role of Stress Echocardiography in Valvular Heart Disease. Curr. Cardiol. Rep. 2022, 24, 1477–1485. https://doi.org/10.1007/s11886-022-01765-7.
16. Altes, A.; Vermes, E.; Levy, F.; Vancraeynest, D.; Pasquet, A.; Vincentelli, A.; Gerber, B.L.; Tribouilloy, C.; Maréchaux, S. Quantification of primary mitral regurgitation by echocardiography: A practical appraisal. Front. Cardiovasc. Med. 2023, 10, 1107724. https://doi.org/10.3389/fcvm.2023.1107724.
17. Wu, Q.; Wang, L.; Li, Y.; Liang, Z.; Li, Q.; Liu, X.; Yin, Y.; Liu, Y.; Hu, Z.; Gao, H.; et al. Prognostic impact of mitral regur-gitation in elderly patients with atrial fibrillation: results from the CABANA trial. Open Heart 2025, 12, e003478. https://doi.org/10.1136/openhrt-2025-003478.
18. https://doi.org/10.3390/jcm9051348.

**Condition being studied** Mitral regurgitation (MR) is one of the most prevalent valvular heart diseases worldwide and represents a major contributor to cardiovascular morbidity and mortality [1]. Its clinical spectrum is highly heterogeneous, encompassing both primary (degenerative) and secondary (functional) forms, with different underlying mechanisms but often overlapping clinical trajectories [2]. Despite advances in imaging and therapeutic strategies, the optimal timing of intervention—particularly in patients with asymptomatic or minimally symptomatic MR—remains a critical and unresolved clinical issue [3].
The natural history of MR is characterized by a prolonged compensated phase, during which patients may remain clinically stable despite progressive structural and functional alterations [4]. However, this apparent stability can be misleading. Subclinical left ventricular (LV) dysfunction, progressive volume overload, and increasing pulmonary pressures may develop before overt symptoms become evident [5]. As a result, reliance on symptom status alone may delay referral for intervention, potentially exposing patients to irreversible myocardial damage and worse postoperative outcomes [6].
In this context, there is a growing need for objective tools capable of detecting early functional impairment and refining risk stratification beyond conventional resting echocardiographic parameters [7,8]. While resting measures such as left ventricular ejection fraction (LVEF), effective regurgitant orifice area (EROA), and regurgitant volume are central to disease grading, they provide only a static snapshot of a dynamic condition [9]. Importantly, MR severity and its hemodynamic consequences are highly load-dependent and may change significantly under physiological stress [10].

References
1. Figlioli, G.; Sticchi, A.; Christodoulou, M.N.; Hadjidemetriou, A.; Amorim Moreira Alves, G.; De Carlo, M.; Praz, F.; Ca-terina, R.; Nikolopoulos, G.K.; Bonovas, S.; et al. Global Prevalence of Mitral Regurgitation: A Systematic Review and

Sonaglioni et al. INPLASY protocol 202640023. doi:10.37766/inplasy2026.4.0023 Downloaded from https://inplasy.com/inplasy-2026-4-0023/

Meta-Analysis of Population-Based Studies. J. Clin. Med. 2025, 14, 2749. https://doi.org/10.3390/jcm14082749.
2. Neema, P.K.; Panidapu, N. The Mechanisms and Pathophysiology of Mitral Regurgitation: A Narrative Review. Ann. Card. Anaesth. 2025, 28, 109–118. https://doi.org/10.4103/aca.aca_221_24.
3. Saeed, M.; Sabanci, R.; Ghnaima, H.; Watat, K.; Shaban, D.; Nader, G.; Banga, S.; Wilcox, M. Navigating Asymptomatic Mitral Regurgitation: Diagnostic Dilemmas and Treatment Strategies. Cureus 2024, 16, e61191. https://doi.org/10.7759/cureus.61191.
4. Delgado, V.; Ajmone Marsan, N.; Bonow, R.O.; Hahn, R.T.; Norris, R.A.; Zühlke, L.; Borger, M.A. Degenerative mitral regurgitation. Nat. Rev. Dis. Primers 2023, 9, 70. https://doi.org/10.1038/s41572-023-00478-7.
5. Pastore, M.C.; Mandoli, G.E.; Dokollari, A.; Bisleri, G.; D'Ascenzi, F.; Santoro, C.; Miglioranza, M.H.; Focardi, M.; Cavi-gli, L.; Patti, G.; et al. Speckle tracking echocardiography in primary mitral regurgitation: should we reconsider the time for intervention? Heart Fail. Rev. 2022, 27, 1247–1260. https://doi.org/10.1007/s10741-021-10100-1.
6. Messika-Zeitoun, D.; Chu, M.W.A.; Bouchard, D.; Le Tourneau, T.; Ternacle, J.; Demers, P.; Guo, L.; Fu, A.Y.N.; Dib, J.C.; Lam, C.; et al. Clinical Presentation and Outcomes After Surgery for Mitral Regurgitation: Real-World Insights From the MITRACURE International Registry. Circulation 2025, 152, 927–938. https://doi.org/10.1161/CIRCULATIONAHA.124.073674.
7. Marchetti, D.; Di Lenarda, F.; Novembre, M.L.; Paolisso, P.; Schillaci, M.; Melotti, E.; Doldi, M.; Terzi, R.; Gallazzi, M.; Conte, E.; et al. Contemporary Echocardiographic Evaluation of Mitral Regurgitation and Guidance for Percutaneous Mitral Valve Repair. J. Clin. Med. 2023, 12, 7121. https://doi.org/10.3390/jcm12227121.
8. Rolando, M.; Elmasry, N.; Gobbi, F.; Moreo, A.; Ajmone Marsan, N.; Carluccio, E.; Fortuni, F. Echocardiographic As-sessment of Patients Undergoing Mitral Valve Repair. J. Cardiovasc. Dev. Dis. 2025, 12, 498. https://doi.org/10.3390/jcdd12120498.
9. Leo, L.A.; Viani, G.; Schlossbauer, S.; Bertola, S.; Valotta, A.; Crosio, S.; Pasini, M.; Caretta, A. Mitral Regurgitation Eva-luation in Modern Echocardiography: Bridging Standard Techniques and Advanced Tools for Enhanced Assessment. Echocardiography 2025, 42, e70052. https://doi.org/10.1111/echo.70052.
10. Duncan, C.F.; Bowcock, E.; Pathan, F.; Orde, S.R. Mitral regurgitation in the critically ill: the devil is in the detail. Ann. Intensive Care 2023, 13, 67. https://doi.org/10.1186/s13613-023-01163-4.

## METHODS

**Search strategy** A comprehensive literature search was independently performed by two investigators to identify studies evaluating the prognostic role of ESE in patients with MR. Studies enrolling patients with both primary (degenerative) and secondary (functional) MR were considered eligible, in order to reflect the broad clinical spectrum of the disease. Electronic databases, including PubMed, Scopus, and EMBASE, were systematically searched from database inception to March 2026.

The search strategy combined controlled vocabulary and free-text terms related to mitral regurgitation and stress echocardiography, including “mitral regurgitation”, “exercise stress echocardiography”, “stress echo”, “exercise testing”, “hemodynamics”, “prognosis”, “outcome”, and “risk stratification”. Search results were screened without restriction to MR etiology. No restrictions were applied regarding language, publication date, or geographic setting.

In addition to the electronic search, the reference lists of all included studies and relevant review articles were manually screened to identify further eligible publications. Any discrepancies between reviewers during the screening process were resolved through discussion and consensus, with the involvement of a third reviewer when necessary.

**Participant or population** Asymptomatic or minimally symptomatic patients with both primary (degenerative) and secondary (functional) MR.

**Intervention** The prognostic role of ESE in patients with primary or secondary MR.

**Comparator** N/A.

**Study designs to be included** Observational Cohort and Cross-Sectional Studies.

**Eligibility criteria** Studies were considered eligible if they met predefined inclusion criteria. Specifically, studies were required to have an observational design, including prospective or retrospective cohort studies, and to include adult patients with primary or secondary (functional) MR. Only studies in which patients underwent exercise-based stress echocardiography were included, ensuring methodological consistency across cohorts. Furthermore, eligible studies were required to report clinical outcomes or prognostic

Sonaglioni et al. INPLASY protocol 202640023. doi:10.37766/inplasy2026.4.0023 Downloaded from https://inplasy.com/inplasy-2026-4-0023/

endpoints, such as mortality, heart failure, mitral valve intervention, or composite cardiovascular events.

To maintain homogeneity in the assessment of functional response, studies employing exclusively pharmacological stress modalities, such as dobutamine stress echocardiography, were excluded unless an exercise component was clearly incorporated.
Additional exclusion criteria were applied to ensure the relevance and quality of the included evidence. Studies including mixed populations of MR without clearly defined cohorts or without extractable outcome data were excluded. Studies were excluded if they did not provide extractable prognostic data, did not report clinical outcomes, or focused solely on diagnostic performance. Moreover, case reports, editorials, conference abstracts, narrative reviews, and preclinical studies were not considered eligible.

**Information sources** Electronic databases, including PubMed, Scopus, and EMBASE, were systematically searched from database inception to March 2026.

**Main outcome(s)** To identify studies evaluating the prognostic role of ESE in patients with MR.

**Additional outcome(s)** N/A.

**Data management** Study selection was performed independently by two reviewers. All retrieved records were initially screened based on title and abstract, followed by full-text assessment of potentially eligible studies according to predefined criteria. Disagreements were resolved through consensus, with arbitration by a third reviewer when required.
Data extraction was conducted using a standardized data collection form developed prior to the review. Extracted information included study characteristics, such as first author, year of publication, study design, and sample size, as well as methodological aspects related to the exercise stress protocol.
Clinical and demographic variables were systematically collected when available, including age, sex distribution, cardiovascular risk factors, and comorbid conditions. Information regarding background medical therapy was also recorded when reported.
Echocardiographic and hemodynamic parameters at rest and during exercise were extracted in detail. These included heart rate and blood pressure, along with comprehensive indices of LV structure and function. In particular, left ventricular systolic performance was assessed through LVEF and LV-GLS, while diastolic function and filling pressures were evaluated using Doppler-derived parameters, including the E/e' ratio.
The severity of MR was characterized using quantitative measures such as EROA and regurgitant volume, as well as categorical grading of MR severity when available. Changes in these parameters during exercise were also recorded to capture the dynamic component of valvular dysfunction .
Pulmonary hemodynamics and right ventricular performance were systematically assessed through sPAP and TAPSE. When available, the TAPSE/sPAP ratio was collected as an index of RV–PA coupling, providing additional insight into ventricular–vascular interaction under stress conditions .
Additional parameters included ventricular volumes, stroke volume, cardiac output, left atrial volume, and indices of functional capacity, such as achieved metabolic equivalents and heart rate recovery.
Clinical outcomes and follow-up data were systematically collected, including duration of follow-up, definition of endpoints, and the main prognostic predictors identified in each study. All extracted data were independently verified by both reviewers, and discrepancies were resolved through re-examination of the original articles.

**Quality assessment / Risk of bias analysis** The methodological quality and risk of bias of the included studies were independently evaluated by two reviewers using the National Institutes of Health (NIH) Quality Assessment Tool for Observational Cohort and Cross-Sectional Studies. This tool assesses multiple methodological domains, including clarity of study objectives, definition of the study population, exposure and outcome assessment, consistency of measurement methods, and appropriateness of statistical analyses. Each domain was rated as "Yes", "No", or "Not Reported" according to predefined criteria.
An overall quality judgment was assigned to each study based on the number of criteria fulfilled and the overall methodological rigor. Disagreements between reviewers were resolved through discussion and consensus. The results of the quality assessment were synthesized both descriptively and through graphical representations to provide an overall view of the risk of bias across studies.

**Strategy of data synthesis** Due to the heterogeneity in study design, patient populations, stress protocols, and endpoint definitions, a formal

Sonaglioni et al. INPLASY protocol 202640023. doi:10.37766/inplasy2026.4.0023 Downloaded from https://inplasy.com/inplasy-2026-4-0023/

quantitative meta-analysis was not performed. Instead, a structured qualitative and descriptive synthesis of the available evidence was conducted.

To provide an overall characterization of the study population, pooled descriptive estimates were derived from study-level data. Continuous variables were summarized as weighted medians with corresponding interquartile ranges, with weighting based on the sample size of each study. When variables were reported as mean and standard deviation, underlying distributions were approximated assuming normality, allowing harmonization across studies.

This approach enabled consistent summarization of demographic, clinical, and echocardiographic variables across heterogeneous datasets. For parameters reported both at rest and during peak exercise, relative changes between conditions were calculated, providing insight into dynamic physiological responses during stress.

Given the observational nature of the included studies, clinical outcomes and prognostic predictors were synthesized qualitatively rather than quantitatively. Predictors were interpreted within their pathophysiological context and grouped into major domains, including MR severity, ventricular function, pulmonary hemodynamics, clinical variables, biomarkers, and functional capacity. The frequency of reporting across studies was also considered to identify the most consistent and reproducible predictors.

No formal pooling of effect sizes, assessment of heterogeneity, or evaluation of publication bias was performed. However, consistency of findings, directionality of associations, and reproducibility across studies were carefully evaluated to support the robustness of the overall interpretation.

All analyses were conducted at the study level. Data processing and aggregation were performed using standard spreadsheet software (Microsoft Excel, Microsoft Corporation, Redmond, WA, USA).

**Subgroup analysis** N/A.

**Sensitivity analysis** N/A.

**Language restriction** No.

**Country(ies) involved** Italy.

**Keywords** mitral regurgitation; exercise stress echocardiography; risk stratification; asymptomatic patients; prognosis.

**Contributions of each author**

Author 1 - Andrea Sonaglioni - Author 1 drafted the manuscript.
Email: sonaglioniandrea@gmail.com
Author 2 - Massimo Baravelli - Supervision.
Email: massimo.baravelli@multimedica.it
Author 3 - Giulio Francesco Gramaglia - The author contributed to the Search Strategy.
Email: giulio.gramaglia@unimi.it
Author 4 - Gian Luigi Nicolosi - The author read, provided feedback and approved the final manuscript.
Email: gianluigi.nicolosi@gmail.com
Author 5 - Michele Lombardo - Supervision.
Email: michele.lombardo@multimedica.it

Sonaglioni et al. INPLASY protocol 202640023. doi:10.37766/inplasy2026.4.0023 Downloaded from https://inplasy.com/inplasy-2026-4-0023/
